# Unperceivable Designs of Wearable Electronics

**DOI:** 10.1002/adma.202502727

**Published:** 2025-05-02

**Authors:** Yijun Liu, Séverine De Mulatier, Naoji Matsuhisa

**Affiliations:** ^1^ Research Center for Advanced Science and Technology (RCAST) The University of Tokyo Tokyo 1538904 Japan; ^2^ Institute of Industrial Science The University of Tokyo Tokyo 1538505 Japan; ^3^ LIMMS/CNRS, Institute of Industrial Science The University of Tokyo Tokyo 1538505 Japan

**Keywords:** e‐textiles, miniaturization, stretchability, transparency, unperceivable electronics, wearable electronics

## Abstract

Wearable smart electronics are taking an increasing part of the consumer electronics market, with applications in advanced healthcare systems, entertainment, and Internet of Things. The advanced development of flexible, stretchable, and breathable electronic materials has paved the way to comfortable and long‐term wearables. However, these devices can affect the wearer's appearance and draw attention during use, which may impact the wearer's confidence and social interactions, making them difficult to wear on a daily basis. Apart from comfort, one key condition for user acceptance is that these new technologies seamlessly integrate into our daily lives, remaining unperceivable to others. In this review, strategies to minimize the visual impact of wearable devices and make them more suitable for daily use are discussed. These new devices focus on being unperceivable when worn and comfortable enough that users almost forget their presence, reducing psychological discomfort while maintaining accuracy in signal collection. Materials selection is crucial for developing long‐term and unperceivable wearable devices. Recent developments in these unperceivable electronic devices are also covered, including sensors, transistors, and displays, and mechanisms to achieve unperceivability are discussed. Finally, the potential applications are summarized and the remaining challenges and prospects are discussed.

## Graphical abstract

Unperceivable wearable technologies seamlessly integrate into everyone's daily life, for healthcare and Internet‐of‐Things applications. By remaining completely unnoticed both visually and tactilely, by the user and others, they ensure medical privacy and allow natural social interactions. Herein are introduced recent strategies employed at material, design, and integration levels to reach unperceivable technologies, whether through transparent materials or strategically hidden devices.

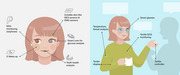

## 1 Introduction

**Figure 1:**
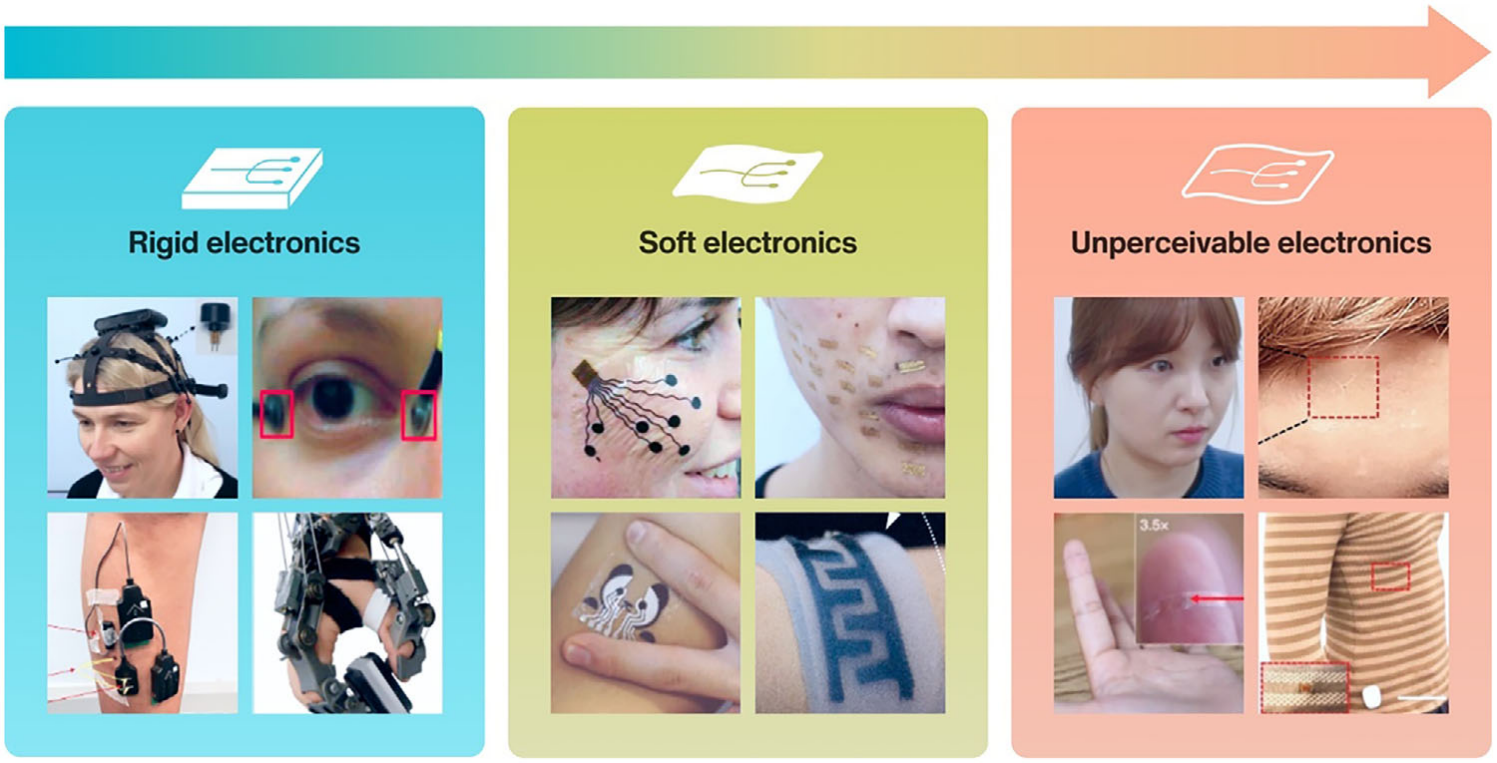
Comparison of various wearable electronics. Rigid electronics. Upper left: dry and wireless EEG system. Reproduced with permission.^[^ ^1^ ^]^ Copyright 2020, Springer Nature. Upper right: electrodes were attached near the inner and outer corners of the eye to measure EOG signals. Reproduced with permission.^[^ ^2^ ^]^ Copyright 2017, Springer‐Verlag Berlin Heidelberg. Lower left: a standard surface sensor was attached to the distal aspect of the right shin to determine foot contact. Reproduced with permission.^[^ ^3^ ^]^ Copyright 2020, Elsevier Ltd. Lower right: photograph of lightweight and versatile hand exoskeleton. Reproduced with permission.^[^ ^4^ ^]^ Copyright 2015, De Gruyter. Soft electronics. Upper left: electrode tattoo array attached to the cheeks. Reproduced with permission.^[^ ^5^ ^]^ Copyright 2018, Springer Nature. Upper right: photograph of nanomesh sensors attached to the face. Reproduced with permission.^[^ ^6^ ^]^ Copyright 2020, Advancement of Science. Lower left: photograph of wearable iontophoretic biosensor device on a printed tattoo platform. Reproduced with permission.^[^ ^7^ ^]^ Copyright 2018, Wiley‐VCH. Lower right: photographs show the flexible, wearable wireless‐charging power system. Reproduced with permission.^[^ ^8^ ^]^ Copyright 2024, American Chemical Society. Unperceivable electronics. Upper left: photographs of a human female subject wearing the soft, smart contact lens. Reproduced with permission.^[^ ^33^ ^]^ Copyright 2021, Advancement of Science. Upper right: sweat collection patch attached to the human forehead. Reproduced with permission.^[^ ^34^ ^]^ Copyright 2022, Elsevier Ltd. Lower left: photography of the pressure sensor on a fingertip. Reproduced with permission.^[^ ^35^ ^]^ Copyright 2020, Springer Nature. Lower right: photograph of wearer wearing a cloth with sensor. Reproduced with permission.^[^ ^36^ ^]^ Copyright 2020, Springer Nature.

Wearable devices advance real‐time health monitoring and human–machine interaction systems. For example, smartwatches and fitness bands monitor users’ heart rate, blood oxygen, and sleep quality. They help users understand their health status and adjust habits. For physical activity, these devices track steps and calorie burn, providing data support for sports enthusiasts and helping them assess their workout efficiency. Furthermore, some wearables feature NFC technology, simplifying the payment process and reducing the burden of carrying items when going out. These wearable devices are based on conventional rigid electronics (**Figure** **1**: rigid electronics).^[^ ^1^, ^2^, ^3^, ^4^ ^]^

The next‐generation wearable devices have been developed using materials as soft and stretchable as skin. Unlike rigid devices, these wearable devices possess flexible and stretchable properties (Figure 1: Soft electronics),^[^ ^5^, ^6^, ^7^, ^8^ ^]^ allowing them to adapt to the body's complex muscle and skeletal structures and adjust their shape freely to follow skin movements during wear. In addition, soft devices can be breathable and sweat‐permeable as induced sweating might lead to skin irritation and discomfort for the user. The enhanced comfort of wear makes the device imperceptible to wearers during use, increasing the acceptance of the technology.^[^ ^9^ ^]^ Various types of soft electronic devices have been realized, including biosensors,^[^ ^10^, ^11^, ^12^, ^13^, ^14^, ^15^, ^16^, ^17^ ^]^ electronic displays,^[^ ^18^, ^19^, ^20^, ^21^ ^]^ and power sources.^[^ ^22^, ^23^, ^24^ ^]^

Another key feature, less often discussed in literature, is the visual impact of wearable devices. For example, electrodes placed on the face provide a wealth of physiological information about the brain's health, but current commercialized electrodes would be difficult to wear every day. Market studies report social influence and perceived aesthetics among the few important parameters for consumers acceptance of wearable technologies.^[^ ^25^, ^26^, ^27^, ^28^ ^]^ For entertainment and Internet‐of‐Things devices, the degree of societal acceptance can affect the willingness to use as some people may be hesitant to be associated with cutting‐edge and expensive new technologies. A notable example occurred in 2013 when Google introduced their first prototype of Google Glass, a pair of smart glasses integrating a camera, head‐ and microphone, and a small display. Their distinctive and bold futuristic design, associated with a high price tag for what was considered a gadget, rapidly rose controversy and cultural criticism around both the technology and its users.^[^ ^29^ ^]^ The consumer version was finally discontinued and Google shifted focus to an enterprise edition. Reactions to new technologies are also heavily influenced by local cultural frameworks. Investigation on consumers’ behavioral intention to adopt wearables among Chinese and Swiss users reveals how perceptions differ from one culture to another.^[^ ^30^ ^]^ Perceived efforts and credibility supported by physicians are reported as factors for acceptance of wearables in Switzerland, whereas external approval and cooperative solutions with healthcare institutions would increase acceptance in China.

For medical applications, which represented more than 25% of the wearable market in 2022,^[^ ^31^ ^]^ unperceivable devices are also highly desirable. Indeed, privacy concerns may rise from obtrusive medical wearables as patients might be reluctant to share their medical conditions with others. Manufacturers specializing in wearable devices for patients with Alzheimer's disease have reported the challenge of having the device to be accepted by the patient.^[^ ^32^ ^]^ One of the main reasons reported for patients’ reluctance was that the device conveyed that they were frail or at risk for health problems. Suggested improvement strategies focused on the visual aspect of the device (removing the branding, reducing to the smallest size possible, hiding it into clothes) to make it the least intrusive. In addition, the psychological discomfort and stress caused by the exposure of personal information can affect the accuracy of monitored signals. Therefore, the way a medical system can seamlessly blend on the patient's appearance will greatly impact both analysis accuracy and success among consumers.

For these reasons, we foresee unperceivable electronic devices (Figure 1: unperceivable electronics)^[^ ^33^, ^34^, ^35^, ^36^ ^]^ as the next‐generation wearable devices. The term “unperceivable wearable” as defined in this review encompasses unobtrusive technologies that have both visual and tactile imperceptibility, specifically designed to remain unnoticed by wearer and surrounding people. Many review papers have discussed developing transparent soft electronics or electronic textiles, with a focus on functionality (for example for transparent smart lens, see‐through tactile screens, combined visual and electrical bioelectronic devices, or transparent electrodes for solar cells) and wearability.^[^ ^37^, ^38^, ^39^, ^40^ ^]^ We would like to review the strategies for unperceivable electronics from the viewpoint of personal and societal acceptances. Various approaches have been explored in recent years including high‐transparency skin electronics,^[^ ^41^ ^]^ miniaturized skin electronics,^[^ ^42^ ^]^ and the integration or concealing into everyday items such as clothing or accessories.^[^ ^43^ ^]^ After an overview of the different strategies explored to achieve unperceivable electronics, we discuss the materials and designs for minimized visibility. Then, we discuss the materials and the design for minimizing the visibility, followed by discussing invisible electronic devices, such as healthcare sensors, antennas, displays, transistors, and energy devices. Next, we introduce strategies explored to integrate and conceal non‐transparent devices inside clothes or everyday accessories. Finally, we address the current limitations of imperceptible electronics and provide insights into their future development directions and potential applications.

## 2 Strategies for Wearable Unperceivable Electronics

**Figure 2:**
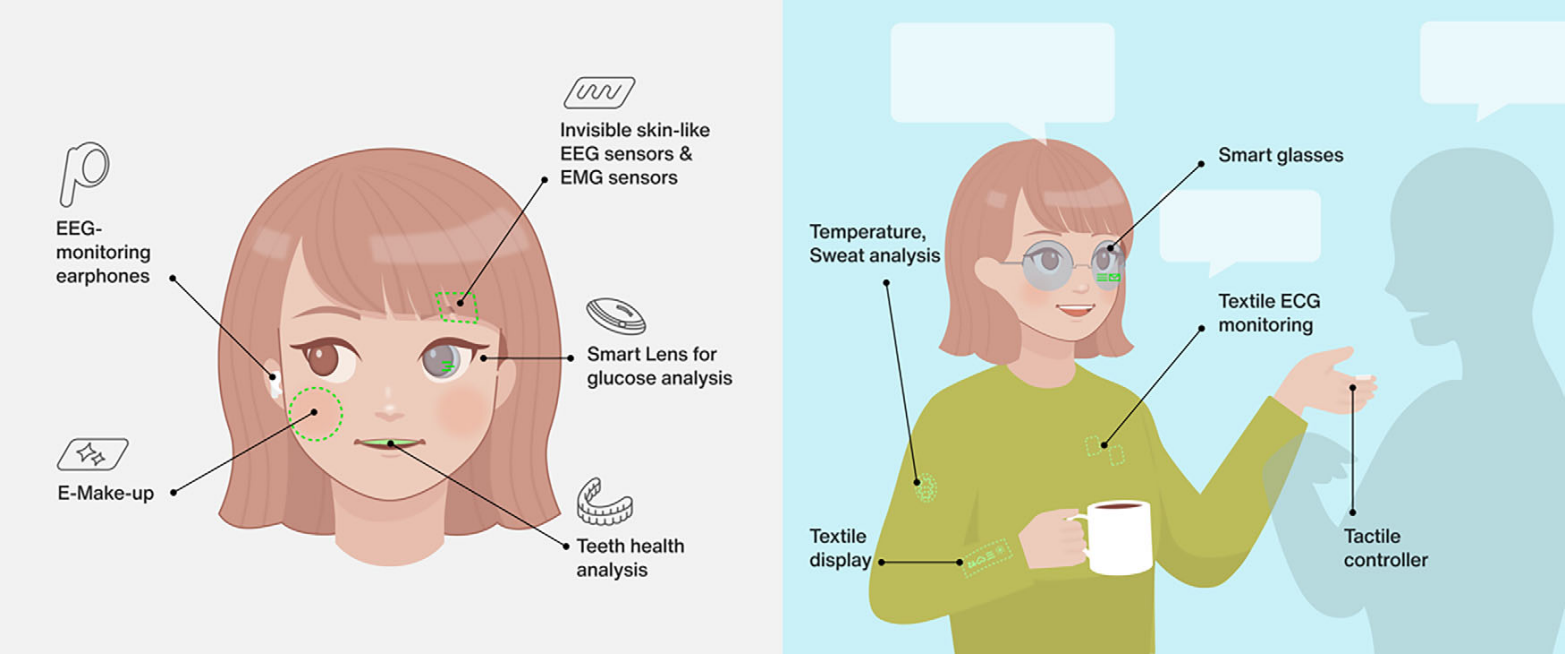
Strategies to realize unperceivable wearables. Various sensors, displays, and user‐machine interfaces are seamlessly integrated in plain sight, in a way that it cannot be perceived by others.

**Figure** **2** summarizes the strategies to realize unperceivability in wearable devices. Applications range from physiological sensing for health monitoring to advanced gadgets for Internet‐of‐Things and human–machine interfaces. Due to their high imperceptibility, they do not alter the user's appearance, allowing discreet use and natural interactions with others.

One strategy for unperceivable devices is to use intrinsically transparent materials, allowing natural skin or support to be seen‐through. This strategy is particularly relevant for applications where the device needs to be placed on exposed parts of the body such as the arms, hands, the face, or the eyes. For example, ultra‐thin and transparent sensors can be integrated on the face to measure electrophysiological signals and monitor various data such as emotions and health, or to be paired with other electronics for human–machine interface. Another example is future electronic make‐up relying on ultra‐thin invisible displays attached to the cheeks. Intrinsically transparent materials and devices are discussed in the first part of this review. Another widely used approach for unperceivable electronics is to integrate them in clothes or inside everyday accessories, such as glasses or oral pads for teeth health monitoring. Finally, non‐transparent materials can be processed in ultra‐miniaturized designs to be embedded on small size devices such as ocular lens for health monitoring.

## 3 Unperceivable Electronic Materials

**Figure 3:**
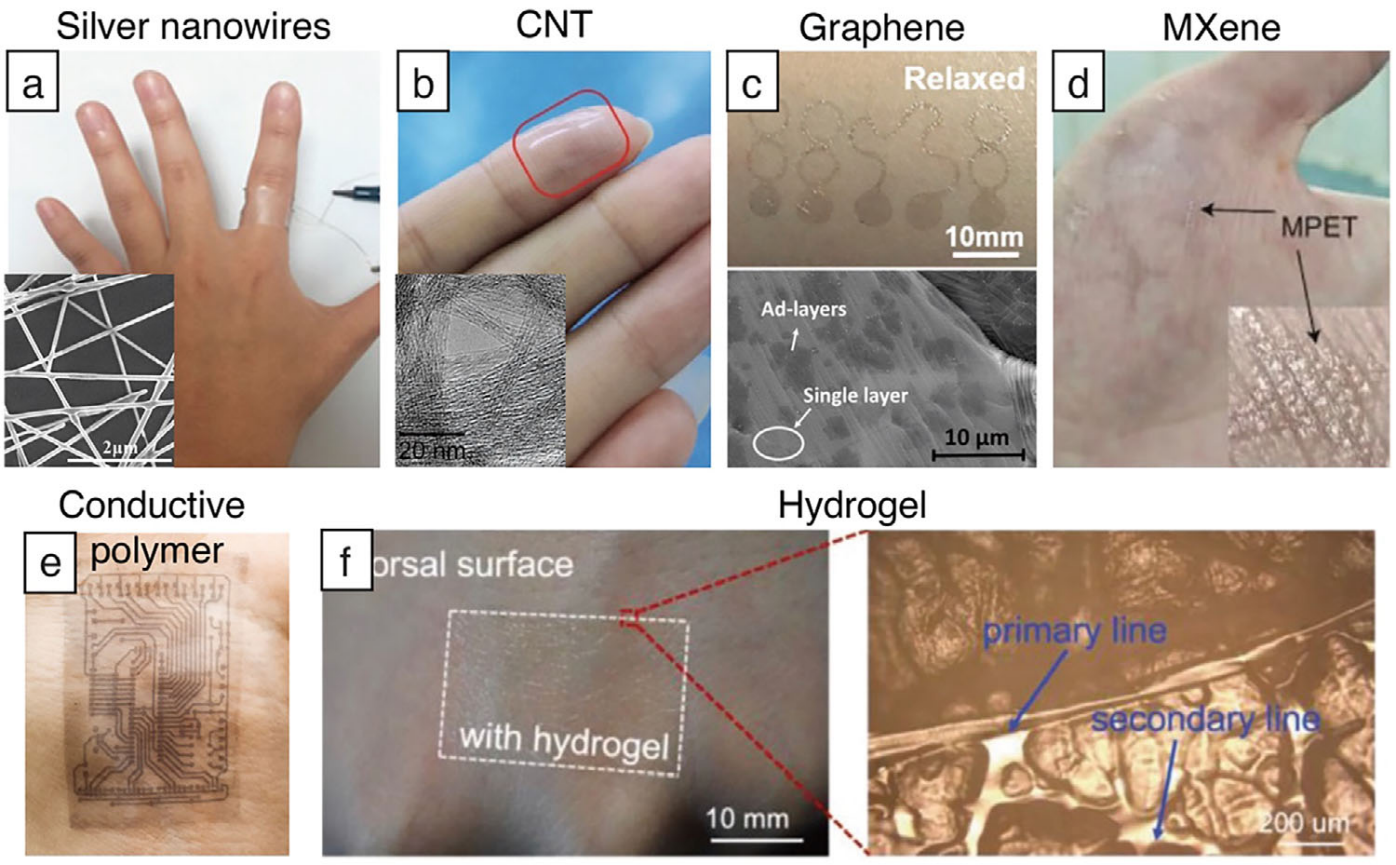
Transparent and soft electronic materials. a) A photograph of the transparent heater on the finger. The inset shows the scanning electron microscope (SEM) image of AgNW after the chemical welding reaction. Adapted with permission.^[^ ^41^ ^]^ Copyright 2023, American Chemical Society. b) The optical image of the stretchable ACNT‐TFTs worn on the finger. Adapted with permission.^[^ ^44^ ^]^ Copyright 2020, Wiley‐VCH. The inset shows the transmission electron microscope (TEM) image of an ultrathin SWCNTs‐TCE on a carbon grid. Adapted with permission.^[^ ^45^ ^]^ Copyright 2023, Wiley‐VCH. c) Graphene electronic tattoo. The inset shows the SEM photo reveals the existence of ad‐layers on monolayer CVD graphene. Reproduced with permission.^[^ ^46^ ^]^ Copyright 2017, American Chemical Society. d) Ultra‐thin MXene film. Reproduced with permission.^[^ ^47^ ^]^ Copyright 2023, Wiley‐VCH. e) Stretchable PEDOT: PSS device placed on the wrist. Adapted with permission.^[^ ^48^ ^]^ Copyright 2022, Advancement of Science. f) Ultrathin hydrogel films. Reproduced with permission.^[^ ^49^ ^]^ Copyright 2022, Wiley‐VCH.

Soft and transparent electrode materials include silver nanowires (AgNWs), conducting polymers, carbon nanotubes (CNTs), graphene, hydrogels, and MXenes (**Figure** **3**). Each material has different optical and electrical properties and realizes various applications to utilize distinct advantages. This part will provide a prospect in unperceivable electronics for each of these materials (**Table** **1**). We especially focus on the works that demonstrate unperceivability.

**Table 1:** Performances of unperceivable electronic materials.

	Sheet resistance	Conductivity	Stretchability	Transparency	Application	Refs.
AgNW	7.5 Ω Sq^−1^	–	N/A	>88%	–	[61]
	8.4 Ω Sq^−1^	–	50%	87.8%	ECG and EMG signal monitoring	[56]
	<50 Ω Sq^−1^	–	>400%	>80%	Human–machine interface	[58]
	6.5 Ω Sq^−1^	–	130%	88%	Electric heater	[41]
	47.4 Ω Sq^−1^	–	N/A	97.4%	Loudspeakers and microphones	[55]
	21.0 Ω Sq^−1^	–	N/A	95%	Touch screen	[54]
	–	10^−2^ S m^−1^	N/A	>99%	Skin temperature and pulse pressure measurements	[112]
	20.8 Ω Sq^−1^	–	–	97.25%	Field effect transistor and heating films	[52]
	18 Ω Sq^−1^	–	N/A	95%	Organic solar cells	[51]
CNT	–	9.5 × 10^4^ S m^−1^	<80%	N/A	Textile generator	[43]
	N/A	N/A	50%	60–80%	Stretchable transistors	[44]
	1.3 Ω Sq^−1^	–	40%	90%	Alternating current electroluminescence devices	[67]
	1800 Ω Sq^−1^	–	85%	50%	Strain sensor	[65]
	–	8.96 S m^−1^	10%	N/A	Differential amplifier	[71]
Graphene	1.3 Ω Sq^−1^	–	80%	<90%	Smart contact lens	[33]
	1994.33 Ω Sq^−1^	–	50%	85%	ECG, EMG, EEG, skin temperature and skin hydration monitoring	[46]
	50.8 Ω Sq^−1^	–	50%	95%	Monitoring of human motion	[77]
MXene	100 Ω Sq^−1^	–	N/A	89%	Bio‐signal monitoring	[47]
	0.5 Ω Sq^−1^	–	N/A	81.6%	Capacitive pressure sensor	[86]
	13.9 Ω Sq^−1^	–	N/A	83.8%	QLED	[85]
Conductive polymer	59 Ω Sq^−1^	3.1 × 10^5^ S m^−1^	800%	96%	LED and FET	[95]
	<1000 Ω Sq^−1^	3.3 × 10^4^ S m^−1^	>150%	<90%	Touch and strain sensor	[96]
	34 398 Ω Sq^−1^		300%	95%	Dielectric elastomer actuators	[113]
	–	5.25 × 10^4^ S m^−1^	100%	N/A	Elastic functional circuits	[99]
	–	6.7 × 10^4^ S m^−1^	20%	N/A	Neural signals monitoring	[100]
	–	8.7 × 10^3^ S m^−1^	32%	N/A	Strain‐humidity sensor	[98]
	–	2.7 × 10^5^ S m^−1^	100%	N/A	EMG and motion signals monitoring	[48]
Hydrogel	150 Ω Sq^−1^	–	200%	92%	Thermal management of skin‐integrated electronics	[49]
	–	0.39 S m^−1^	975%	96.2%	EEG and EMG monitoring and pressure detection	[105]
	–	9.26 × 10^−3^ S m^−1^	100%	>50%	Monitoring of human motion	[106]
	–	5.33 × 10^2^ S m^−1^	>1200%	89%	Strain sensor	[109]

### 3.1 AgNWs

AgNWs are 1D nanomaterials with diameters below 100 nm. Compared to other soft transparent conductors, AgNWs exhibit excellent electrical conductivity (≈1000–10 000 S cm^−1^) and superior optical transparency. Due to their flexibility, AgNWs can easily attach to irregular surfaces such as skin and remain unaffected by bending. Further, the network's structure as well as the nanowires’ diameter and length can be modified to meet specific application requirements. Compared to other transparency materials, their unique conductivity, transparency, and flexibility make them particularly suitable as flexible displays and touchscreens.^[^ ^50^ ^]^

In the development of unperceivable electronics utilizing AgNWs, achieving high conductance, transparency, and flexibility is important. Welding treatments can bridge the junctions between nanowires, reducing the contact resistance while maintaining optical transparency. Chemical welding technique can be used to bond AgNWs with a layer of aluminum‐doped zinc oxide (AZO), through capillary‐induced welding and secondary growth of AZO.^[^ ^51^ ^]^ This process reduces the junction resistance within the AgNW network, achieving a low sheet resistance of 35.2 Ω Sq^−1^ and a high transmittance of 91.6%. Irradiation welding techniques using intense pulsed light (IPL) have been explored to locally heat AgNW junctions.^[^ ^52^ ^]^ This method melts and welds the junction without damaging flexible substrates. It can successfully reduce the sheet resistance to 20.8 Ω Sq^−1^; while, achieving a transmittance of 97.25%. Further, embedding AgNWs within a composite substrate made of silk fibroin, cellulose, and silk‐poly(vinyl)alcohol (S‐PVA) not only welds the AgNWs but also enhances their biocompatibility. The AgNWs undergo UV‐ozone treatment, resulting in a structure with high transparency, flexibility, and a low sheet resistance of 15 Ω Sq^−1^ on the S‐PVA substrate.^[^ ^53^ ^]^ A further reduction in sheet resistance to 6.5 Ω Sq^−1^, coupled with excellent bending stability (Figure 3a) is achieved through chemical welding of AgNWs at room temperature. This process increases electrical conductivity without altering the density of AgNWs.^[^ ^41^ ^]^ These AgNW layers can be worn on the finger as highly transparent electrodes, showing significant stability enhancement under various environmental and acidic conditions.

Different strategies were explored to control the structure of the AgNWs network and tune its electrical and optical properties. The nanowires could be aligned in one selected direction with a bar‐coating process using a Meyer rod (a rod with a slit pattern) over an AgNWs solution. An orthogonal array of AgNWs was obtained by successively bar‐coating in two perpendicular directions, maximizing the substrate coverage while minimizing wires overlap.^[^ ^54^ ^]^ The resulting array achieved a better ratio of conductivity to transparency (21.0 Ω Sq^−1^ at 95.0%) compared to random network of AgNWs (21.0 Ω Sq^−1^ at 90.4%). Bar‐coating is also an interesting approach as the coated surface depends on the rod's size, allowing large‐scale fabrication. Using this method, transparent and conductive hybrid nanomembranes of orthogonal AgNWs array embedded in polymer matrix with enhanced conductivity (47.4 Ω Sq^−1^) and transparency (97.4%) were demonstrated.^[^ ^55^ ^]^ Skin‐attachable nanomembrane loudspeakers and microphones were fabricated based on this process. Another approach to pattern ultrathin AgNW network is evaporation‐induced self‐assembly.^[^ ^56^ ^]^ This method effectively organizes the distribution of AgNWs without undesirable aggregation. The result is an ultrathin electrode with high transparency (87.8%) and low sheet resistance (8.4 Ω Sq^−1^) that can withstand multiple bending cycles and maintain conductivity over 60% stretchability.

Despite the flexibility and transparency achieved, AgNWs are susceptible to breakage when subjected to excessive or repeated stretching.^[^ ^57^ ^]^ Enhancing the stretchability can be achieved by modifying the physical structure of the substrate. Stretchable transparent electrodes have been developed using a combination of kirigami structures and laser ablation technology.^[^ ^58^ ^]^ This approach allows stretchability over 400%; while, maintaining high optical transparency. Electrodes for super‐stretchable heaters and electrophysiological sensors for continuous health monitoring were demonstrated based on this technology and were integrated in human–machine interfaces to control quadcopter drones.

Using simultaneously conjugated electrospinning and electrospray techniques, polyurethane nanofiber sensor yarns embedded with AgNWs have been developed.^[^ ^59^ ^]^ These yarns successfully integrate strain and pressure‐sensing capabilities into wearable textiles. They exhibit high stretchability (up to 160%), high gauge factor (GF) (≈1010), and pressure sensitivity of up to 16.7 N^−1^. In personalized healthcare and human–machine interfaces, wearable devices utilize AgNWs to make solution‐processed, transparent thermal radiation shields, which are designed based on theoretical optical models.^[^ ^60^ ^]^ The produced textiles have low mid‐infrared emissivity and high visible light transmittance, adaptable to any shape and size.

Long‐term stability is another challenge for AgNWs‐based films as wearable electrodes are typically subjected to electrical bias, mechanical folding, and high humidity. AgNWs are integrated with zinc oxide nanoparticles (ZnONPs) using an in situ solution processing method to address this issue.^[^ ^61^ ^]^ This combination results in a nanocomposite with enhanced adhesion and mechanical strength, improving durability under various load conditions; while, maintaining excellent electrical and optical properties.

### 3.2 Carbon Nanotube (CNT)

CNTs film primarily forms through van der Waals forces, which are weak attractions between the tubes to improve the aggregation and strength. These forces help maintain conductivity and mechanical properties, even under extreme conditions.^[^ ^62^, ^63^ ^]^ Their semiconducting or metallic electrical properties make them highly suitable for use in manufacturing electronic and optoelectronic devices, such as transistors and logic gates.^[^ ^64^ ^]^ When dispersed as single or few‐layer structures on a transparent substrate, CNTs maintain high transparency, making them unperceivable when worn on the skin.

Single‐walled carbon nanotubes (SWCNTs) are renowned for their unique mechanical strength, electrical conductivity, and chemical stability. However, their high surface energy promotes bundles formation in solutions, which restricts their practical applications. Using purpurin, a naturally occurring dye molecule, in aqueous solutions, helps exfoliate SWCNTs.^[^ ^45^ ^]^ The solution‐based method produces bucky papers and transparent conductive electrodes that feature high‐quality networks rich in metallic SWCNTs. These materials offer robust mechanical strength and high conductivity, coupled with a high flexibility and transparency that allows seamless adhesion to various surfaces (Figure 3b).

Traditional patterning methods such as ink printing and photolithography can damage the unique properties of CNTs. A novel dry transfer printing technique uses CNTs blocks as stamps, allowing CNT patterns to be directly printed onto flexible substrates^[^ ^65^ ^]^ and achieving a GF of up to 9960 at 85% strain, with high stretchability (>200%) and durability (>5000 cycles). The line width limiting resolution of 150 µm makes this printing method a great candidate for unperceivable electronics. Laser‐assisted patterning and dry deposition techniques also enable the assembly of vapor‐phase CNTs into flexible devices without the need for photolithography to develop photodetector arrays.^[^ ^66^ ^]^ These arrays offer excellent uniformity, wear resistance, environmental stability, and a significant broadband light response, with a high responsivity of 44 AW^−1^ and a detectivity of 1.9 × 10^9^ Jones. High‐throughput direct patterning of CNT aerosols avoids using adhesives and complex processing steps.^[^ ^67^ ^]^ The patterned electrodes exhibit a low sheet resistance of just 1.3 Ω Sq^−1^ and a high transmittance of 90%. Further, color electroluminescent devices can be fabricated on these patterned electrodes, enhancing their aesthetic appeal when worn.

CNTs‐based unperceivable electronics can also be integrated into our everyday clothes. For example, a carbon nanotube fiber (CNTF) has been developed by twisting four CNT films wrapped with acrylic fibers.^[^ ^43^ ^]^ A stretchable thermoelectric module was demonstrated from woven CNTFs. Textile generators can be developed using these thermoelectric modules that enable sufficient alignment with the heat flow direction. At a temperature difference of 44K, these textile generators achieve a peak power density of 70 mW m^−2^ and exhibit excellent stretchability, maintaining the output power at 80% strain. This development advances unperceivable electronics and enhances the efficient utilization of thermal energy, expanding the application of CNTs in wearable power‐generating devices. Further, integrating CNTs directly into fabrics eliminates problems such as skin irritation and discomfort associated with traditional CNT electrodes that rely on adhesives. By wrapping CNTs around cotton and spandex yarns using textile techniques, adhesive‐free, highly conformable, and washable dry electrodes are fabricated for biosensing applications.^[^ ^68^ ^]^ These electrodes closely conform to the skin and can measure high‐fidelity electrocardiogram (ECG) signals. In addition, different thicknesses of TiO₂ layers are deposited on the CNT surface to add a fashionable element to wearable CNT electronic devices.^[^ ^69^ ^]^ Utilizing the principles of physical optics, including thin‐film interference and surface reflection, this approach deviates from the inherent black color of CNTs to achieve various colors. The TiO_2_ coating enhances durability, withstanding 2000 wash cycles without fading and maintaining its color stability under intense UV light for over 10 months.

CNTs are ideal materials for high‐performance transistors due to their outstanding semiconductor properties and high carrier mobility.^[^ ^70^ ^]^ Flexible transistors normally employ inorganic dielectric layers such as Al_2_O_3_ and HfO_2_, which can restrict mechanical deformation. Metallic carbon nanotubes (M‐CNT) are utilized as electrodes and semiconducting carbon nanotubes (S‐CNT) as channels in the development of high‐performance and stretchable CNT transistors.^[^ ^44^ ^]^ A polyvinyl alcohol (PVA) hydrogel is used as the gate dielectric, topped with an amorphous SiO_2_ layer deposited via low‐temperature plasma‐enhanced chemical vapor deposition (PECVD). The resulting transistors exhibit excellent mechanical deformability and stable electrical performance under 50% strain. This high transparency significantly reduces the visibility of the electrodes, making them unperceivable even when worn on the finger (Figure 3b). Ultra‐thin, highly flexible dual‐gate CNT transistors, with a thickness of less than 180 nm, are suitable for the high‐fidelity acquisition of biological signals.^[^ ^71^ ^]^ These transistors conform closely to biological surfaces without drawing attention. Using DNA templates, the spacing of CNT arrays is precisely controlled to achieve ultra‐dense alignments of less than 10 nm.^[^ ^72^ ^]^ This structural approach facilitates further miniaturization of electrode sizes without sacrificing conductivity and performance, paving the way for the development of future minimal and unperceivable electronics.

### 3.3 Graphene

Graphene is a 2D material consisting of a single layer of carbon atoms; while, CNTs are graphene rolled into a tubular structure. Both materials are composed of sp^2^ hybridized carbon atoms arranged in a honeycomb‐like structure, giving them similar electronic and mechanical properties.^[^ ^73^ ^]^ Graphene's high surface area and electrical conductivity make it an ideal material for the fabrication of high‐performance supercapacitors for fast charging and discharging applications.^[^ ^74^, ^75^ ^]^ Further, it achieves especially high transparency with a transmittance of up to 97.7%, enabling devices that can be attached to exposed skin. Graphene's adaptability to various environments and oxidation resistance are beneficial for developing unperceivable electronics close to the body or intended for long‐term use.

Graphene has enabled the development of unperceivable and highly sensitive strain sensors capable of detecting subtle human movements. Encapsulating single‐crystal hexagonal graphene (SCG) within a PVA substrate protected the sensor from issues such as wrinkles, grain boundaries, and additional layers.^[^ ^76^ ^]^ This sensor showed high sensitivity, with a GF of 16.9, significantly surpassing traditional graphene film sensors in performance; while, maintaining high transparency. It can accurately detect various subtle human movements, including finger motions, micro‐vibrations from sounds, and heartbeats. A wearable piezoresistive pressure sensor has been developed using a stable network of reduced graphene oxide self‐wrapped around copper nanowires. It exhibits high sensitivity (0.144 kPa^−1^), a wide sensing range (0.1–15 kPa), rapid response time (<150 ms), and excellent long‐term stability (over 1000 cycles).^[^ ^77^ ^]^

Graphene's high carrier mobility (3544 cm^2^ V^−1^ s ^−1^) enables the development of graphene field‐effect transistors (GFETs) for unperceivable electronics. These transistors can detect in real‐time matrix metalloproteinase‐9, a biomarker for ocular surface inflammation found in tears.^[^ ^33^ ^]^ This capability is integrated into a smart contact lens designed to monitor and treat chronic ocular surface inflammation, offering real‐time, non‐invasive monitoring and thermotherapy. Further, an ultra‐flexible and transparent wearable nanosensor based on graphene field‐effect transistor has been developed for the detection of body fluid‐biomarker.^[^ ^78^ ^]^ This sensor detects L‐cysteine in undiluted human sweat with a detection limit of 0.022 µm and remains structurally and electrically stable after 100 extensive deformations. Thanks to its high transparency (98%) and flexibility, the sensor can be placed on the eye for tear analysis without affecting vision.

Ultra‐thin, highly conformable electronic tattoos for long‐term biometric sensing have been developed using a “wet transfer, dry patterning” method to produce sub‐micron thick graphene electronic tattoos (Figure 3d).^[^ ^46^ ^]^ The tattoos are over 40% stretchable and maintain about 85% optical transparency. This type of graphene electronic tattoo reduces device costs and enables high‐precision, continuous monitoring of electrooculography (EOG), electroencephalography (EEG), and facial electromyography (EMG) without causing psychological discomfort to the wearer.^[^ ^79^ ^]^ Using roll‐to‐roll production technology, large‐area piezoelectric thin films have been developed. These films incorporate piezoelectric lead zirconate titanate (PZT) nanoparticles and graphene nanosheets aligned in the thickness direction to develop transparent, flexible loudspeakers.^[^ ^80^ ^]^ These speakers can seamlessly integrate into fabrics without visible components or other bulky add‐ons.

### 3.4 MXene

2D materials are atomically thin and extend in a 2D plane, granting them flexibility due to their single‐atom thickness.^[^ ^81^ ^]^ Unlike graphene and CNTs, which are composed purely of carbon, MXenes are derived from layered structures of transition metal carbides or nitrides, obtained through chemical etching, and commonly include metals such as titanium and vanadium.^[^ ^82^, ^83^ ^]^ This multi‐element composition of MXenes provides additional opportunities to modulate their chemical properties. For example, changing the constituent metals or intercalating other elements between layers can adjust electrical conductivity, electrochemical activity, or thermal stability.^[^ ^84^ ^]^ Due to their exceptional properties, MXenes show great potential in developing wearable biosensors with tunable electrochemical sensitivity. In addition, MXenes exhibit excellent electromagnetic interference shielding, making them well‐suited for use in smart clothing and devices.

Although highly conductive transparent MXene electrodes hold great potential, their application in unperceivable electronics is limited by high sheet resistance, which results from flake connectivity issues and surface roughness. A hybrid electrode has been developed by combining a high conductivity AgNW network with solution‐processed MXene flakes, termed MXene‐AgNW (MXAg) hybrid electrode.^[^ ^85^ ^]^ It exhibits a sheet resistance of ≈13.9 Ω Sq^−1^ and transparency of 83.8%, making it an ideal electrode material for high‐performance transparent quantum dot light‐emitting diodes (QLEDs). A biomimetic, hierarchically interwoven MXene mesh with AgNWs has been developed to overcome the trade‐offs among high transparency, low sheet resistance, and excellent mechanical flexibility in unperceivable electronics.^[^ ^86^ ^]^ This mesh achieves a low sheet resistance of 0.5 Ω Sq^−1^ and transparency of 81.6%, enabling the unperceivable monitoring of human bio‐signals.

A breakthrough in reducing environmental noise interference has been made with the development of a 20‐nm thick MXene film crosslinked with poly(3,4‐ethylenedioxythiophene):poly(styrene sulfonate) (PEDOT:PSS). This film enables high‐fidelity detection of electrophysiological signals and is compatible with imaging techniques without causing magnetic or optical artifacts (Figure 3d).^[^ ^47^ ^]^ The ultra‐thin structure and the transparency of the materials allow unperceivability. In addition, using the good solubility of MXene in water can help develop intraocular pressure sensors. Integrating patterned MXene electrodes into contact lenses has demonstrated exceptional sensitivity, measuring 7.483 mV mmHg^−1^, and allows for non‐invasive monitoring of intraocular pressure when cleverly combined with contact lenses.^[^ ^87^ ^]^

The exceptional photothermal conversion efficiency of MXene has been utilized in ocular photothermal therapy devices. By spray‐decorating a transparent layer of Ti_3_C_2_T*_x_* MXene onto commercial contact lenses, effective improvements in vascular blood flow within the eye have been achieved.^[^ ^88^ ^]^ Further, MXene‐decorated textiles have been developed for monitoring human motion and managing heat.^[^ ^89^ ^]^ This is achieved by decorating nanoporous polyethylene (nanoPE) textiles with Ti_3_C_2_T*_x_* MXene to develop MXene/nanoPE textiles.^[^ ^90^ ^]^ This fabric demonstrates excellent active solar heating, reaching up to 73.5 °C and Joule heating capabilities (55 °C at 5 V).

Long‐term usability and adaptability to various environments are essential for wearable electronic devices. Appling a thin polymer layer onto solution‐processed MXene significantly enhanced its anti‐oxidation properties, maintaining stability for over 7 days even under conditions of 70 °C and 50% humidity.^[^ ^91^ ^]^

### 3.5 Conductive Polymer

PEDOT:PSS (poly(3,4‐ethylenedioxythiophene):polystyrene sulfonate) is a conductive polymer composite material with excellent biocompatibility, composed of two parts: PEDOT and PSS. PEDOT is a conjugated conductive polymer known for its high electrical conductivity, but it is inherently insoluble in water. PSS acts as a dopant and dispersant for PEDOT, stabilizing its molecules to form aqueous solutions or dispersions.^[^ ^92^ ^]^ PEDOT:PSS is a highly transparent material with a transmittance above 90%. Although its conductivity is lower than that of metals or carbon materials such as graphene and CNTs, it is adequate for many low‐power electronic devices. Further, as PEDOT:PSS can conduct both electrons and ions, it exhibits very low electrochemical impedance. This makes it highly suitable for monitoring neural signals and other electrophysiological signals with a high signal‐to‐noise ratio.^[^ ^93^, ^94^ ^]^

PEDOT:PSS is renowned for its high electrical conductivity and transparency, but lacks of intrinsic stretchability in comparison to elastomers or hydrogels. This limitation restricts its use in wearable electronics, which require substantial mechanical performances. The addition of ionic additives and stretch enhancers to PEDOT:PSS films has significantly improved their conductivity and stretchability.^[^ ^95^ ^]^ These modified films achieve a high conductivity of over 4100 S cm^−1^ at 100% strain. Patterned on styrene–ethylene–butadiene‐styrene (SEBS) substrate, these films exhibit only a light blue color. A stretchable conducting polymer composed of PEDOT:PSS doped with lithium bis(pentafluoroethanesulfonyl)imide (LiBETI) is developed by spin‐coating the solution onto a thermoplastic polyurethane (TPU) substrate, facilitating the development of wearable sensor arrays.^[^ ^96^ ^]^ The stretchability of PEDOT: PSS is significantly enhanced by aligning the mechanical properties of the conducting polymer with the TPU substrate. This polymer maintains transparency on the skin and can be patterned using nanosecond UV laser ablation adding versatility to wearable device designs.

Applying PEDOT: PSS on fabrics can be challenging due to the poor surface wettability of fabrics, which limits uniform film formation when using traditional solution processes. Oxidative chemical vapor deposition is utilized to develop thickness‐controlled, patterned polymer films that preserve the inherent advantages of the fabric.^[^ ^97^ ^]^ This approach has successfully been used to monitor physiological signals such as blood pressure and respiratory rate through the fabric. Screen printed PEDOT:PSS/natural rubber latex composites on fabrics show enhanced stretchability and electrical performance, resulting in flexible, durable sensors.^[^ ^98^ ^]^ These sensors exhibit a GF of 123.8 with excellent linearity, a humidity response time as low as 0.72 s, and a recovery time of 0.85 s. They can be designed into attractive patterns on fabric, enabling health monitoring without affecting the sensor's performance.

The device size can be reduced by effectively patterning PEDOT, thereby minimizing the visibility of wearable electronic devices. A novel single‐layer optical lithography technique called PhotoAssist has been proposed. It can directly pattern PEDOT through UV‐triggered solubility modulation without the need for photoresist and removal steps. With this technique, transistors with a channel length of 2 µm can be fabricated, achieving a density of ≈42000 transistors per cm^2^ and a conductivity of 3.15 ± 0.68 kS m^−1^.^[^ ^99^ ^]^ A topological supramolecular network has been developed to make patterns with small feature sizes (Figure 3e). The network can combine high mechanical robustness with excellent electrical conduction (≈2700 S cm^−1^).^[^ ^48^ ^]^ In addition, laser‐induced phase separation of PEDOT:PSS (LIPSP) technology utilizes continuous‐wave lasers to efficiently separate PEDOT from PSS. High‐conductivity PEDOT micro‐patterns achieve a conductivity of 670 S cm^−1^ and good stability in water.^[^ ^100^ ^]^ A low‐cost, disposable paper‐based ammonia sensor has been developed by combining PEDOT: PSS with iron (III) compounds for enhanced sensitivity and selectivity.^[^ ^101^ ^]^ This sensor is ten times smaller than traditional sensors and performs well in humid environments. Its compact size enables integration into masks or direct placement inside of the nostrils. In developing unperceivable electronics with PEDOT:PSS, maintaining moisture stability is also a critical challenge. The performance of electrodes and electrolytes degrades significantly when exposed to atmosphere. PEDOT:PSS films susceptible to degradation and delamination in humid conditions can be stabilized with the integration of a PEG layer to form a photo‐patternable hybrid film.^[^ ^102^ ^]^ This enhancement allows the film to maintain higher humidity stability for over 10 days, with a photolithography resolution as low as 2 µm. The flexibility and patternability of the film make it suitable for embedding in fabrics to monitor physiological signals such as respiratory rate.

### 3.6 Hydrogel

Hydrogels are 3D network polymers formed by chemical cross‐linking, contributing to softness and mechanical properties similar to human tissue.^[^ ^103^ ^]^ While their electrical conductivity is generally lower than materials such as AgNWs and CNTs, hydrogels can reach higher stretchability. They are also highly biocompatible, suitable for use as long‐term wearable bio‐signal monitoring electrodes and show a high and colorless transparency. The large amount of water contained in hydrogels confers a texture and appearance similar to biological tissues. This characteristic makes them highly suitable for developing electronic skin (e‐skin). Further, the unique rheological properties of hydrogels make them ideal for use in 3D bioprinting.^[^ ^104^ ^]^

Hydrogels offer promising alternatives to conventional rigid electronic devices due to their excellent biocompatibility and stretchability.^[^ ^105^, ^106^, ^107^ ^]^ Hydrogels are designed to use minimal energy during normal cyclic loading; while, exhibiting high toughness and fatigue resistance. An electronic skin system was developed based on a polyacrylamide (PAM) hydrogel modified with vinyl hybrid silica nanoparticles with MXene and polypyrrole nanowires (PpyNWs), achieving significant toughness with low hysteresis.^[^ ^108^ ^]^ The hydrogel provides a robust and elastic substrate for the electronic skin, which offers a 2800% working range and a fast response of 90 ms. It demonstrates excellent elasticity with a recovery time of 240 ms and maintains outstanding reproducibility over 5000 cycles. The hydrogel's transparency and conformability allow it to seamlessly integrate on the body, remaining unperceivable on skin. Tough, fatigue‐resistant, and self‐healing ionic hydrogels containing self‐healable elastic nanonetworks have been developed.^[^ ^109^ ^]^ These hydrogels demonstrate ultra‐high strain and pressure sensitivity (GF of 4.58 and 0.56 kPa^−1^, respectively), a broad working range (0–1200% for strain and 0–250 kPa for pressure), and excellent repeatability, with nearly unchanged output signals over 500 stretch and compression cycles. With 89% transparency, these hydrogels’ function as unperceivable sensors for monitoring human motion, including joint movements, pulse rate, and subtle actions such as speaking.

Highly ionic hydrogels have been developed by incorporating charge‐rich polyether into a natural polysaccharide network.^[^ ^105^ ^]^ These hydrogels exhibit 975% stretchability and 96.2% optical transparency, and are highly sensitive with a GF of 3.26 for stretching and 7.34 for compression. Their skin comfortability and self‐adhesive quality ensure excellent adherence to the skin, enhancing the accuracy of biosignal collection. Wearable electronics require high performance in varying external environments. A freeze‐resistant, highly stretchable, and biocompatible organohydrogel has been developed using a binary solvent system of water and ethylene glycol, enhanced with charged functional groups.^[^ ^110^ ^]^ The hydrogel acts as a sensor, maintaining ≈99% transparency, super stretchability (6185%), and toughness (9.2 MJ m^−3^). It can quickly respond to signals even at −40 °C. Its high transparency makes it virtually unnoticeable when used as electrodes. In monitoring biosignals, water exposure can damage bioelectrodes and affect their performance. A paintable hydrogel has been developed to address this issue. It transitions from liquid to gel upon application to the skin, enhancing adaptability and maintaining performance in moist conditions.^[^ ^111^ ^]^ Applying the hydrogel directly on skin or on fabrics enables in situ fitting to any surface morphology. This technique results in electrodes with lower contact impedance and more stable signal.

For long‐term wear, it is important that electrodes are breathable to prevent skin damage and signal artifacts caused by the skin's unnatural functioning. Ultrathin hydrogel films produced by cold lamination offer this breathability, allowing the skin to function naturally and without irritation.^[^ ^49^ ^]^ These films are durable enough for long‐term wear (Figure 3f). They are ultra‐thin, highly transparent, and can seamlessly attach on skin. Finally, wearable devices made from non‐degradable materials pose environmental challenges. An eco‐friendly solution employs edible cassava hydrogel, with viscoelastic and self‐healing properties, to develop biodegradable skin‐like iontronic devices.^[^ ^106^ ^]^ This hydrogel demonstrates excellent cycle repeatability and is capable of monitoring subtle human motions, such as micro‐expressions and speech recognition.

### 3.7 Summary of Unperceivable Materials

Here, we summarize the materials for unperceivable electronics (Table 1). AgNWs’ high conductivity, transparency, and flexibility make them ideal for developing unperceivable electronics, especially large‐scale displays and touch sensors. CNTs show high mechanical, chemical, and thermal stability. They also exhibit semiconducting and metallic electrical properties, making them widely used in high‐performance transistors and other semiconductor devices. Graphene also shows high stability for long‐term use. MXene's conductivity and chemical properties can be adjusted by changing the constituent metals or intercalating other elements, enabling versatile and selective sensors. Conductive polymers such as PEDOT:PSS can conduct both electrons and ions, resulting in very low electrochemical impedance. These properties make them ideal materials for monitoring electrophysiological signals. Hydrogels are highly biocompatible and can be comfortably worn on the skin. They are particularly useful in unperceivable electronic devices due to their colorless and high transparency properties.

## 4 Unperceivable Devices

### 4.1 Sensor

Unperceivable sensors integrate health monitoring seamlessly into daily activities without causing discomfort to the wearer. These sensors require high transparency and flexibility, with a low Young's modulus to ensure comfortable wear and minimal mechanical irritation on skin. Selecting suitable materials is critical. This section addresses the challenges of unperceivable sensors in practical applications, such as signal sensitivity, reliability, and long‐term durability. Recent research progresses and future directions for unperceivable sensors are also discussed.

#### 4.1.1 Strain Sensors

Strain sensors monitor muscle activity, joint flexion, or breathing rates in health applications^[^ ^118^ ^]^ by measuring deformations such as stretching, compression, and bending. Strain sensors are typically classified into three types: piezoelectric, piezoresistive, and capacitive. Piezoelectric strain sensors are based on the piezoelectric effect, where mechanical stress leads to deformations within the sensor, causing internal polarization and opposite charges generation at the sensor's electrodes.^[^ ^119^ ^]^ The amount of charges being proportional to the applied force, voltage measurement allows precise measurement of stress. Piezoresistive strain sensors operate by detecting changes in electrical resistance when the material is mechanically deformed, either stretched or compressed.^[^ ^120^ ^]^ Capacitive strain sensors measure strain through changes in capacitance, consisting of two parallel conductive plates separated by a dielectric material.^[^ ^121^ ^]^ Applied external pressure alters the distance between the plates or the characteristics of the dielectric, affecting the capacitance.

**Figure 4:**
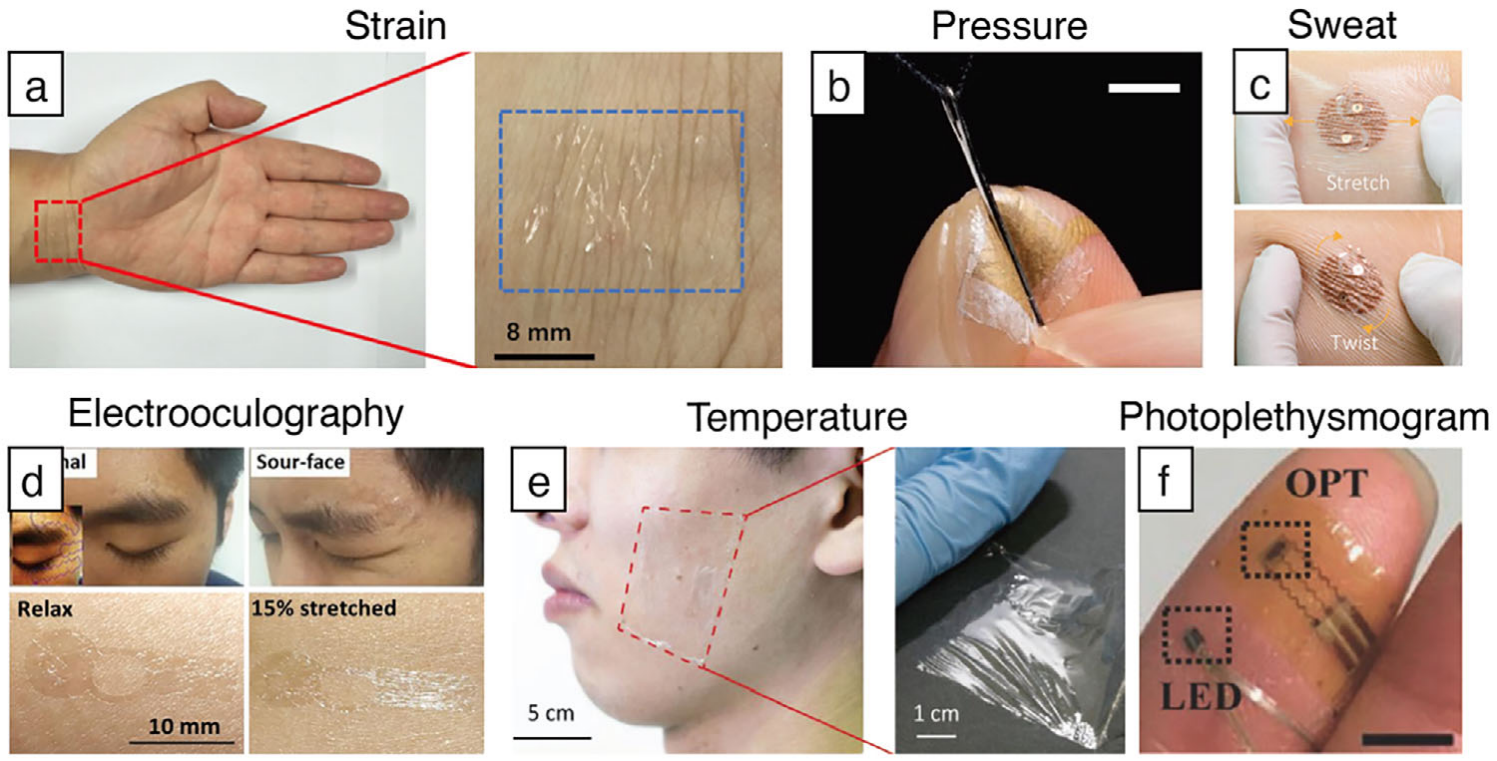
Unperceivable sensors. a) Photograph of a strain sensor adhered to the wrist. Reproduced with permission.^[^ ^114^ ^]^ Copyright 2020, American Chemical Society. b) Nanomesh pressure sensor for monitoring finger manipulation. Reproduced with permission.^[^ ^115^ ^]^ Copyright 2020, Advancement of Science. c) Photographs of the sensor attached to human skin and under stretch and twist conditions. Reproduced with permission.^[^ ^116^ ^]^ Copyright 2021, Advancement of Science. d) Imperceptible electrooculography graphene sensor worn around human eyes. Adapted with permission.^[^ ^79^ ^]^ Copyright 2018, Springer Nature. e) The ion gel thin‐film sensor is attached to the cheek. Reproduced with permission.^[^ ^117^ ^]^ Copyright 2022, Wiley‐VCH. f) Flexible organic/inorganic hybrid near‐infrared photoplethysmogram sensor. Reproduced with permission.^[^ ^42^ ^]^ Copyright 2017, Wiley‐VCH.

Transparent conductive materials such as CNTs,^[^ ^122^ ^]^ hydrogels,^[^ ^123^ ^]^ and graphene^[^ ^76^ ^]^ were employed to develop strain sensors that do not interfere with natural skin color, making them ideal for unperceivable wearable strain sensors. A transparent, skin‐conformal resistive strain sensor was developed for an accuracy‐improved, gesture recognition system based on the fusion of visual and somatosensory data.^[^ ^124^ ^]^ The invisibility of the sensors was primordial to remain inconspicuous in the photographs. Single‐walled CNTs were used as the sensing component, combined with a polydimethylsiloxane (PDMS) layer and a hydrogel adhesive layer. Different concentrations of single‐walled CNTs were tested for the best combination of high transparency and reliable strain sensing performance. The resulting sensors showed high transparency (83% at 550 nm), stretchability of 100%, and sensitivity high enough to capture subtle human hand gestures. The final visual/sensory data fusion approach could achieve over 96.7% of recognition accuracy even in the dark. A simple water‐bath pulling method was developed to achieve ordered array structures of AgNWs for highly sensitive and transparent strain sensors.^[^ ^125^ ^]^ The maximum GF reached 84.6 at a strain of 30%. An ultra‐miniaturized sensor was developed based on a single centimeter‐scale silicon nanowire with a diameter of only 600 nm, embedded on a pre‐strained PDMS substrate (**Figure** **4**a).^[^ ^114^ ^]^ The detection range of the sensor could be adjusted with the level of pre‐strain, allowing it to accurately sense joint bending movements as well as subtle actions like swallowing. The diameter, smaller than that of spider silk, made the sensor inconspicuous during wear. A strain sensor featuring a nanomesh structure exhibited a stretch stiffness of 76.1 ± 5.0 mN mm^−1^, which is lower than that of skin, ensuring that the device did not cause discomfort or a foreign body sensation to the wearer, especially during long‐term wear.^[^ ^126^ ^]^ The ultra‐soft and ultra‐thin characteristics of the sensor ensured it adhered closely to the skin, minimizing visibility and interference with the signal.

#### 4.1.2 Pressure Sensors

Pressure sensors and strain sensors operate on similar principles. Pressure sensors can also be designed based on piezoelectric, piezoresistive, and capacitive techniques to meet various application needs. They are widely used in medicine for monitoring vital signs such as pulse and blood pressure, ensuring safe and precise treatment for patients needing internal and external pressure monitoring.^[^ ^127^, ^128^ ^]^ Pressure sensors are also used to measure weight, and detect human pulse and grip postures.^[^ ^129^, ^130^ ^]^

Flexible capacitive pressure sensors are typically designed with a vertical sandwich structure of electrodes on both sides of a soft dielectric layer. The low signal drift provides high accuracy of measurement over long‐term use, making them ideal for human–computer interaction and health monitoring.^[^ ^131^ ^]^ However, the sensitivity of these sensors is limited due to the restricted compressibility of the soft dielectric layer. Micropores or surface microstructures are often introduced into the dielectric layer to improve compressibility and enhance sensitivity. These microstructures can strongly scatter light, resulting in high haze and reduced transparency of the dielectric layer. One strategy to avoid this effect is to use materials with similar optical properties. Filling a polyvinylidene fluoride (PVDF) membrane with an ionic liquid that matches the refractive index of PVDF achieves a balance between high sensitivity and transparency, with a final optical transmittance up to 94.8%.^[^ ^132^ ^]^ This method holds the potential for developing transparent components such as batteries, further supporting unperceivable wearable devices. Using a low density of microstructures in a transparent dielectric is another strategy to promote the invisibility of the device. A transparent and highly sensitive pressure sensor based on the tunneling effect was demonstrated, using sea urchin‐like hollow carbon spheres with dimensions of ≈600 nm in diameter and 80 nm in length of spikes, dispersed in a transparent PDMS matrix. The sensor exhibited an ultrahigh sensitivity of 260 kPa^−1^ at 1 Pa and theoretical sensing density of 2718557 cm^−2^.^[^ ^35^ ^]^ The microscale size of the fillers and their low density (<1.5 wt%) in PDMS ensured the high transparency of the device.

In addition to being visually unperceivable, sensors should also be unperceivable to the touch, especially when placed on sensitive areas such as the fingertips. For example, measuring grip force requires sensors directly attached to the fingertips, which can impair natural skin sensations. To address this issue, an ultra‐thin pressure sensor has been developed using a nanomesh structure (Figure 4b).^[^ ^115^ ^]^ This sensor maintains its functionality under repeated pressure and friction, demonstrating high mechanical durability with minimal sensory interference. Volunteer sensory experiments confirm its minimal impact on tactile sensation when monitoring finger pressure.

Some flexible pressure sensors can cause a pronounced foreign body sensation during long‐term wear due to mismatched Young's modulus or excessive thickness, failing to achieve tactile imperceptibility. A mechanically and biologically skin‐like elastomer has been developed to enhance the compatibility of bio‐integrated electronic devices with soft human skin.^[^ ^133^ ^]^ The material's toughness is enhanced through a dual‐network structure consisting of physical and covalent crosslinks; while, maintaining a low modulus. The skin‐like elastomer‐based pressure sensor mimics the mechanical properties of human skin. This achieves significant advancements in biocompatibility and biodegradability.

#### 4.1.3 Sweat Sensors

Sweat contains a wealth of physiological information, such as electrolytes, metabolites, amino acids, and hormones, which can reflect a person's health status.^[^ ^134^ ^]^ Particularly during physical activity, the levels of sodium and potassium in sweat can indicate whether a person is experiencing dehydration. Analyzing metabolites, such as lactic acid, can also assess muscle fatigue. Flexible sweat sensors are devices that can be discreetly adhered to the skin to detect analytes in sweat, providing a comfortable wearing experience.^[^ ^135^ ^]^ Ideally, they should remain unperceivable to the wearer during exercise and not draw attention from others.

Ultrathin and transparent sweat sensors have been developed to achieve unperceivable monitoring.^[^ ^136^ ^]^ In addition, sensors covered under cartoon images look like stickers, making them obvious but not recognized as monitoring devices during wear.^[^ ^137^ ^]^ Existing wearable electronics often require connection to external devices for power and data display, limiting autonomy and mobility. Using high‐throughput screen printing technology, custom stretchable composite inks have been used to develop an independent, stretchable epidermal sweat sensing platform.^[^ ^138^ ^]^ This platform integrates stretchable batteries and displays, showing data directly without external connections. It operates independently, worn on the finger with direct readouts visible on the device. An electrochromic display integrated into the electrochemical sensor platform shows fatigue data instantly to cater to high‐intensity sports.^[^ ^139^ ^]^ This sensor platform remains stable during sports activities without any wired or wireless connection to external devices. Real‐time monitoring of the concentration of various electrolytes or metabolites in sweat assists athletes in adjusting their training intensity and preventing overtraining. The device also exhibits excellent stretchability, conforming well to skin movements even during intense activities; thus, ensuring comfort and minimizing any foreign body sensation for the wearer. A new sensing platform with universal molecular recognition ability has been developed to overcome the issue of selective analytes in current sweat analysis devices (Figure 4c).^[^ ^116^ ^]^ Using flexible SERS‐active plasmonic metasurfaces, the device can successfully monitor changes in drug concentrations within the body and obtain individual drug metabolism profiles. The sensor's design focuses on comfort with its flexible and scalable structure conforming to the skin. The ultra‐thin sensor can be designed in personalized shapes, improving its aesthetics.

#### 4.1.4 Electrophysiological Signals Sensors

Flexible electrode devices adapt to complex body parts thanks to their softness and can monitor precise electrophysiological signals. These signals reflect changes in cell membrane potentials through variations in current and voltage, such as ECG,^[^ ^140^ ^]^ EEG,^[^ ^141^ ^]^ EOG,^[^ ^142^ ^]^ and EMG^[^ ^143^ ^]^ for heart, brain, eyes, and muscle activity, respectively. Monitoring these electrophysiological signals allows for early detection of health abnormalities and diseases, enabling timely intervention. The human face contains over 40 muscles and is one of the body's most complex muscular structures.^[^ ^144^ ^]^ Visible electrodes on the face can affect the wearer's appearance and induce a psychological burden, affecting the physiological signals being measured. Unperceivable electrodes provide aesthetic improvements and adhere closely to the skin without causing a foreign body sensation, enabling more accurate signal monitoring.

Despite the widespread use of flexible electrodes in healthcare and human–machine interfaces, their thickness often impedes sweat evaporation, leading to discomfort and a noticeable sensation. A “super tattoo” only 2‐µm thick has been developed by mimicking the skin's collagen fiber network and gel matrix.^[^ ^145^ ^]^ This ultra‐thin structure not only reduces light refraction and scattering, enhancing optical integration with the skin, but also provides excellent breathability and comfort during long‐term wear. Especially for the most sensitive skin around the eyes, ultra‐soft sensors are essential. A graphene electronic tattoo sensor developed using “wet transfer, dry etching” techniques, and measuring just 500 nm thick, adheres directly to the skin around the eyes (Figure 4d).^[^ ^79^ ^]^

In addition to transparent materials, specifically tuned coloring materials can be used to make unperceivable devices. A novel organohydrogel was developed using collagen sourced from natural skin.^[^ ^146^ ^]^ The skin‐like color and ultra‐thin properties make it nearly unperceivable when worn on the skin, facilitating unnoticeable use in daily life. Further, this hydrogel maintains biocompatibility and characteristics of tissues even under extreme temperatures ranging from −196 °C to 100 °C.

A soft electronic skin (e‐skin) system was demonstrated, integrating flexible circuits and sensors into a single device platform.^[^ ^147^ ^]^ This integration involves a high‐performance transistor array only sub‐micron thick, keeping the device's volume and weight minimal. The electronic skin system is specifically designed for optimal adaptability and functionality on various body parts. This design allows the e‐skin to precisely conform to complex human contours, continuously monitor, and respond to external stimuli without causing discomfort.

#### 4.1.5 Temperature Sensors

A temperature sensor is a monitoring device that responds to temperature changes. During the COVID‐19 pandemic, measuring body temperature became an important method to identify potential infections.^[^ ^148^ ^]^ Research is focusing on developing flexible and stretchable temperature sensors that adhere closely to the skin, minimizing the influence of external factors on measurements.

Traditional film sensors made from nanomaterials often face limitations in transparency, flexibility, and breathability due to complex manufacturing methods. A simpler spray‐coating technique is employed on 25‐µm thick PDMS film, using ion gels containing various ionic liquids (ILs), to form layers sensitive to temperature and humidity (Figure 4e).^[^ ^117^ ^]^ The use of ion gels enhances breathability and when integrated with the PDMS film, it allows the sensors to function in various environments; while, remaining nearly unperceivable. Some specific applications also require that the sensors adapt to non‐conventional environments, such as underwater. For example, divers can monitor bio‐signals underwater to maintain awareness of their physical state, but traditional sensors often degrade underwater. A new type of ion‐gel sensor was developed to address this issue.^[^ ^149^ ^]^ With ultra‐thin design and high transparency, such design ensures continuous and reliable sensing on skin or other surfaces without impacting visual aesthetics. Further, its self‐adhesive properties enable easy attachment to the skin without the need for additional adhesives and without causing skin damage.

An ultra‐flexible transparent temperature sensor was fabricated using laser‐direct writing of AgNWs.^[^ ^150^ ^]^ It can remain virtually unperceivable when worn due to its high transparency and excellent conformability. This characteristic is achieved by ensuring a tight integration of AgNWs with the PVDF substrate and a smooth surface during the film fabrication process.

#### 4.1.6 Other Sensors

Flexible organic/inorganic hybrid near‐infrared photoplethysmogram (PPG) sensors are widely used to assess an individual's daily physiological status, including cardiovascular parameters such as heart rate^[^ ^151^ ^]^ and blood pressure.^[^ ^152^, ^153^ ^]^ Commercially available PPG sensors typically employ traditional integrated circuit technology, which is large and rigid. This limits the accuracy of physiological data collected during physical activity or in environments with strong light interference. A hybrid organic phototransistor (OPT) with inorganic light‐emitting diodes (LEDs) has been developed using a high dielectric constant gate dielectric and bulk heterojunction (BHJ) active layer, achieving low voltage operation (<3 V) (Figure 4f).^[^ ^42^ ^]^ This sensor exhibits excellent mechanical flexibility and chemical stability, can be directly transferred onto human skin, and is as small as a fingertip. Using an ultrathin polymer encapsulation layer and positioning the OPT at the neutral mechanical plane, the sensor maintains good air stability and mechanical flexibility, adhering closely to the skin for physiological signal monitoring, making it compatible with long‐term monitoring.

Giant magnetoresistive (GMR) sensors utilize the giant magnetoresistive effect to measure magnetic field strengths. However, current printable magnetic sensors are limited in their ability to withstand mechanical strain when bent and typically only detect strong magnetic fields. This restricts their use on the skin due to potential health risks associated with continuous exposure to high magnetic fields. A new stretchable and printable GMR sensor has been developed that operates in low magnetic field environments and features excellent mechanical stability and sensitivity.^[^ ^154^ ^]^ By using poly(styrene‐butadiene‐styrene)(SBS) as an adhesive, which has Young's modulus similar to the bare skin, the sensor can adhere to the skin without causing discomfort.

### 4.2 Other Devices

Wearable smart systems are generally composed of several of the following elements: sensors measuring various health and environmental values; electronic circuits composed of transistors for data processing; an interfacing device displaying information; antennas to wirelessly collect data, control the system, or even power the device; and untethered power source such as battery or energy harvesting device. This section discusses different materials and fabrication techniques explored to make these devices transparent; while, maintaining electrical performances for good functionality.

#### 4.2.1 Antennas

**Figure 5:**
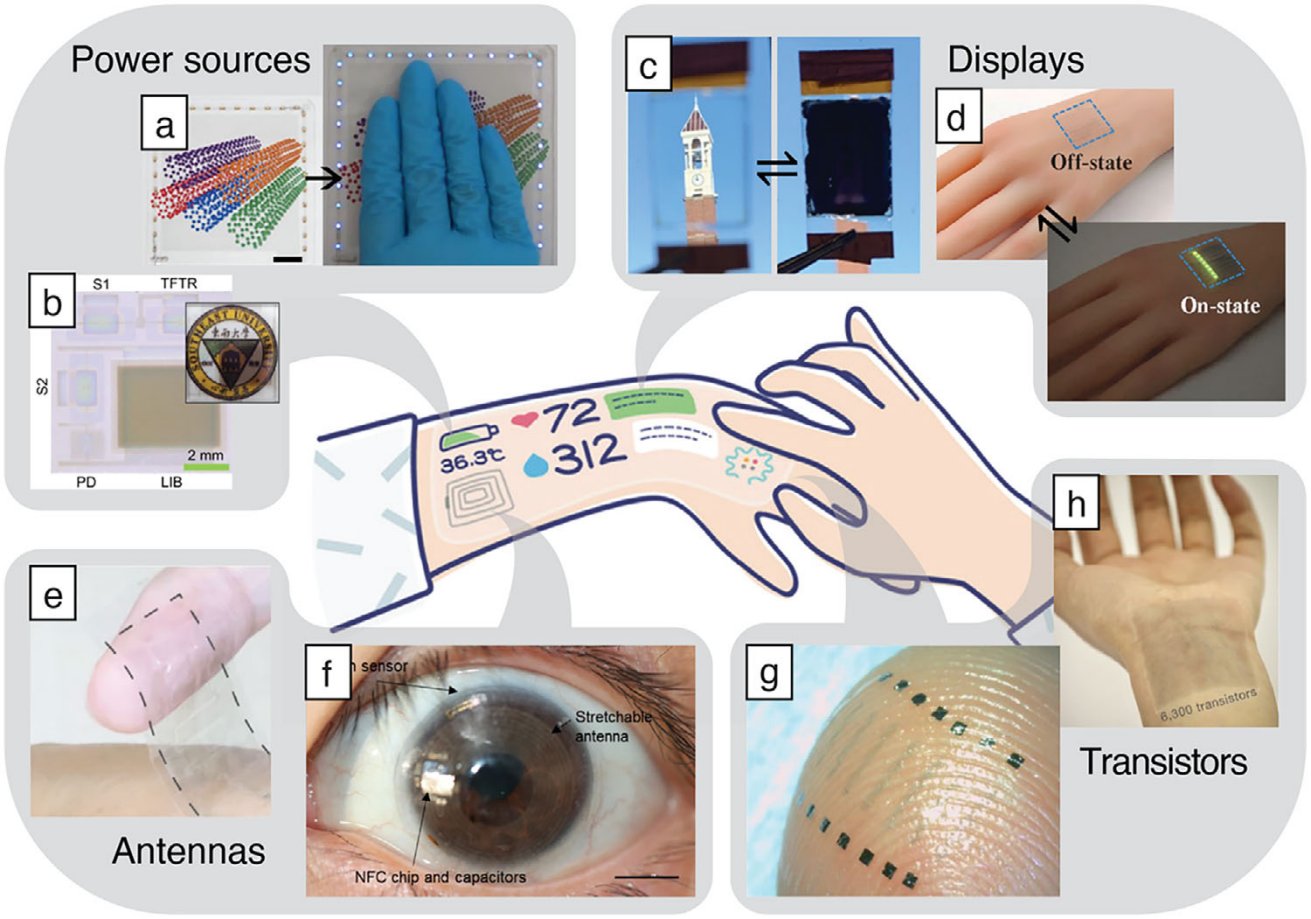
Elements of wearable smart systems with invisibility based on intrinsically transparent electronic materials. Schematic of the next‐generation wearable systems. Reproduced with permission.^[^ ^9^ ^]^ Copyright 2022, John Wiley and Sons. Power sources: a) transparent and stretchable triboelectric nanogenerators integrated with chip LEDs. Adapted with permission.^[^ ^155^ ^]^ Copyright 2020, Elsevier Ltd. b) Transparent microsystem integrating a battery. The inset shows the system see‐trough transparency. Adapted with permission.^[^ ^156^ ^]^ Copyright 2023, Springer Nature. Displays: c) electrochromic display under bleached and colored states. Adapted with permission.^[^ ^157^ ^]^ Copyright 2024, American Chemical Society. d) Transparent and conformable OLED on a hand prosthesis. Adapted with permission.^[^ ^158^ ^]^ Copyright 2023, Elsevier. Antennas: e) ultrathin and transparent RF sensor based on silver nanowires. Reproduced with permission.^[^ ^159^ ^]^ Copyright 2024, Springer Nature. f) Transparent antenna mounted on a smart lens designed for intraocular pressure monitoring. Scale bar: 0.5 cm. Reproduced with permission.^[^ ^160^ ^]^ Copyright 2021, Springer Nature. Transistors: g) semi‐transparent transistors arrays on‐skin. Reproduced with permission.^[^ ^161^ ^]^ Copyright 2018, Springer Nature. h) Transistor arrays with transparency and high density of integration. Reproduced with permission.^[^ ^162^ ^]^ Copyright 2021, Springer Nature.

Wearable systems generally require wireless tools to communicate with external mobile devices such as phones, smart watches, or other computational system. Antennas also enable battery‐free operation in size‐critical applications such as smart lenses. For smart lenses^[^ ^33^, ^160^, ^163^ ^]^ and smart glasses,^[^ ^164^ ^]^ antennas with high optical transparency are particularly relevant as they allow wireless communication without obstructing the wearer's view, as shown in **Figure** **5**f. A key challenge in creating transparent antennas is that electrical conductivity and optical transparency are typically inversely correlated. Therefore, larger antenna designs are employed to counterbalance high electrical resistivity, which is not ideal in most wearable applications. A transparent near‐field‐communication (NFC) antenna was achieved by combining spray‐coated AgNWs on top of a random network of electrospun silver nanofibers, allowing local bridging between Ag nanofibers.^[^ ^160^ ^]^ The film reached average values of sheet resistance and transparency (at 550 nm) of 0.3 ± 0.05 Ω Sq^−1^ and 73 ± 2.2% respectively. The final antenna design of nine coils with 300‐µm width and 50‐µm space could be integrated on the lens without obstructing the wearer's view, allowing standard NFC communication with a smartphone at 10 mm of distance. Also based on Ag nanomeshes, other research works focused on room‐scale network with transparent radio‐frequency (RF) tags for wireless and battery‐free ambient intelligence applications.^[^ ^159^ ^]^ Using a AgNWs mesh, they could extend the working range from 8 to 15.2 m, which is within 84.4% of the operating range of standard non‐transparent tags based on copper, by increasing the density of the nanowires network. These RF tag sensors, shown in Figure 5e, achieve over 90% transparency, making them ideal for integration into living environments without compromising aesthetics or functionality. Liquid metal meshes were also largely explored for stretchable antennas that can freely deform in wearable applications.^[^ ^165^, ^166^ ^]^ With the adequate grid patterning, liquid metal mesh films reach high ratio of optical transparency to electrical conductivity. A soft coplanar antenna was demonstrated with a mesh film of gallium–indium eutectic alloy in PDMS encapsulation.^[^ ^167^ ^]^ The pattern lines had width and spacing of 50 and 500 µm respectively, resulting in a Square resistance of 0.0456 Ω Sq^−1^ for optical transmission of 72%. The resulting antenna had a measured peak gain of 3.38 dBi, average efficiency of 61% at 3 GHz, and operation frequencies covering multiple channels for 5G communications.

#### 4.2.2 Semiconductor Devices

Flexible thin‐film transistors (TFT) enable integrated circuits in rollable displays, flexible sensors, and electronic skins.^[^ ^168^ ^]^ Conventional TFT is composed of a semiconductor layer, a gate insulator layer, and three terminals (drain, source, and gate electrodes). Flexible and transparent transistors require that each layer shows soft mechanical properties and high optical transmission. The electrodes, in addition to flexibility and transparency, should present superior conductivity and low contact resistance with the semiconductor layer. Different materials explored to obtain such electrodes were discussed previously of this section.

Regarding the semiconducting layer, performance metrics are high on/off current ratio, high field‐effect mobility, and small threshold voltages. One strategy to obtain transparent semi‐conducting materials with these performances is to use inorganic materials with large optical bandgap such as transition metal oxides (>3 eV) and nitrile films (>5 eV). Transparent resistive switching RAM (random access memory) using ZnO^[^ ^169^ ^]^ and AlN^[^ ^170^, ^171^ ^]^ are investigated paired with ITO electrodes on glass. Final memory devices reach over 80% of transmission in the visible range. Though they are built on rigid glass, these memories can be integrated in see‐through devices such as interface displays, smart glasses, and other applications where transparency is key. Methods to fabricate metal oxides with soft mechanical properties include building 2D films via atomic layer deposition process,^[^ ^172^ ^]^ low‐temperature solution processes,^[^ ^173^, ^174^, ^175^ ^]^ or using semiconductor materials in their nanostructure form. Figure 5g depicts a sub‐150‐nm, all‐organic field‐effect transistor fabricated using a fully solution‐based approach. This ultrathin design enables the device to conformally adhere to nonplanar surfaces such as human skin and bend to a radius smaller than 1 µm.^[^ ^162^ ^]^ Fully transparent and flexible nanowire transistors (NWT) were developed based on In_2_O_3_ and ZnO nanowire active channels.^[^ ^176^ ^]^ Associated with atomic layer deposition of Al_2_O_3_ as gate insulator, ITO and IZO as contacts, on PET substrate, they obtained optical transmissions of ≈82% and ≈83% in the 350–1350 nm wavelength range, for In_2_O_3_ NWTs and ZnO NWTs, respectively.

Full imperceptibility on skin requires not only invisibility but also skin‐like stretchability. Semiconducting materials with both stretchability and good carrier mobility are particularly challenging to obtain as properties associated with high carrier mobilities (namely rigid chains in π‐conjugated polymers and high degrees of crystallinity) and deformability are opposed. Several structural strategies were developed to address this challenge. First, a blending approach using nanowires of organic semiconductor in an elastomer matrix was explored for improved stretching stability. Nanowires of poly(3‐hexylthiophene) (P3HT) organic semiconductor were blunt with PDMS in a ratio of 10 wt% of P3HT in PDMS.^[^ ^177^ ^]^ The resulting layer achieved stretchability up to 100% of strain with a charge mobility 100 times faster than for pristine P3HT in the same stretched conditions due to the formation of percolated networks of the semiconductor NWs with a high crystallinity and a large aspect ratio. Moreover, thanks to the transparency of both P3HT and PDMS, the final device reached a high transparency over 90% across the visible spectrum. Second, nanoconfined polymer structures achieved transistor devices with a stretchability of 100%; while, maintaining semiconducting properties similar to the ones of silicon (charge‐carrier mobility of 0.98 cm^2^ V^−1^ s^−1^).^[^ ^161^, ^178^, ^179^ ^]^ These performances are attributed to the connectivity between confined nanofibrils maintaining the charge transport mobility even under strain; while, the interface with soft elastomer delays the onset of crack formation. An intrinsically stretchable transistor array based on the nanoconfinement effect is depicted in Figure 5h.^[^ ^161^ ^]^ Due to the quasi‐homogeneous all‐SEBS based structure, the transistor array displays exceptional stretching capabilities up to 100% strain in both directions; while, maintaining a charge‐carrier mobility of 0.98 cm^2^ V^−1^ s^−1^. Reported arrays include an active matrix for sensory arrays and analogue and digital circuit elements.

#### 4.2.3 Displays

Displays are widely used and intuitive user‐machine interfaces. For wearable technologies, the human body is an interesting platform to integrate soft displays: the large surface of skin enables wider displaying areas than rigid smart watches; the skin‐like mechanical properties and strap‐free adhesion to skin allows a broad range of locations, keeping sensitive health information hidden from others and accessible only to the user. Wearable transparent displays are mainly based on two technologies: electrochromic materials,^[^ ^157^, ^180^, ^181^ ^]^ with their color or opacity reacting to applied voltage through reversible chemical reactions; and electroluminescent materials,^[^ ^107^, ^182^, ^183^, ^184^, ^185^ ^]^ emitting light in response to electrical current.

One advantage of electrochromic (EC) displays is their typically low range of operating voltages from 1.5 to 5 V DC, making the device safer to use on skin. Their efficiency as display relies on the on/off color ratio (or on/off opacity contrast): the display should be completely transparent when not in use to remain unperceivable and show a strong enough color when needed to properly display information. Researchers have tuned the absorption band gap of EC aromatic polymers to reach full transmittance in the visible region.^[^ ^157^ ^]^ By adding meta‐conjugated linkers, they achieve nearly 100% transmittance in the neutral state and a high absorption in the oxidized state, reaching an optical contrast exceeding 93%, as shown in Figure 5c. An EC film containing polymers with different absorption spectrum, such as Fe(II) and Ru(II)‐based metallosupramolecular polymers, can successfully display multiple colors, from purple to orange to transparent, by varying the potential application time.^[^ ^181^ ^]^

Depending on the application, self‐emitting electroluminescent (EL) displays can be desired. EL materials include organic‐based emissive layers,^[^ ^158^, ^185^, ^186^ ^]^ inorganic‐based phosphors,^[^ ^182^, ^187^ ^]^ perovskites,^[^ ^188^ ^]^ and quantum dots.^[^ ^85^ ^]^ As for every other device, optical properties of the conducting electrodes are crucial for the overall transparency of the device. Figure 5d shows a transparent, skin conformable organic EL device.^[^ ^158^ ^]^ By coupling EL polymers with highly deformable and transparent anodes and cathodes, namely high‐conductive PEDOT:PSS/SWCNTs as the ultra‐flexible transparent anode and 8‐Quinolinolato lithium/Al/Ag/ZnS as the ultra‐thin laminated transparent cathode, researchers developed an EL device with high average transmittance up to 74.7% in the visible range (400–700 nm) and high mechanical flexibility with skin‐like conformability. One drawback of EL technology is the high operating voltages are not always safely compatible with on‐skin wearable applications. The safety on‐skin could be improved by using high‐k dielectric materials for EL capacitors, lowering operating voltages and alternating currents. The high‐k dielectric matrix is obtained by adding a small amount of non‐ionic fluorinated surfactant to a high‐κ poly(vinylidene fluoride) (PVDF)‐based fluoroelastomer.^[^ ^187^ ^]^ The dielectric‐EL layer is composed of phosphor microparticles mixed in the high‐k dielectric material, reaching *κ* value between 10 and 27. The device turns on at an alternating voltage of 23 V and a frequency inferior to 1 kHz, ensuring safe operation for human–machine interactions. By using transparent materials, optical transparencies of the electrode and the dielectric are in the ranges 94–100% and 80–97% in the visible light wavelengths. Another alternating current EL device is developed based on a luminescent layer made of chlorinated barium titanate and phosphor in polydimethylsiloxane (PDMS), sandwiched between transparent electrodes composed of AgNWs, cellulose nanocrystals with II crystalline allomorph (CNC II), and Triton X‐100 modified PDMS.^[^ ^182^ ^]^ CNC II and Triton X‐100 are specifically chosen to maintain or enhance optical transmission of AgNWs and PDMS. Final device reaches a transmittance of 79.5% at 550 nm.

#### 4.2.4 Energy Devices

Wireless powering is crucial for wearable applications and can be achieved in three ways: as we discussed previously, timely wireless powering can be implemented through antennas; energy storage can be integrated directly into the device; or an energy harvesting layer can be used to convert external stimuli into electrical supply. Transparent energy storage systems are currently one of the limitations to fully transparent wearables due to the challenge of designing electrodes that combine optical transmittance, high conductivity, and high capacitance. Indeed, the charge density being proportional to thickness, there is a trade‐off between electrical properties and transparency. Transparent batteries with flexible^[^ ^189^, ^190^, ^191^ ^]^ and stretchable^[^ ^192^ ^]^ mechanical properties were developed. Strategies to obtain transparent batteries include patterning methods of the electrode in a grid‐structured design that cannot be perceived by eye.^[^ ^192^, ^193^ ^]^ By combining sub‐100 µm lines grid‐electrodes with a transparent gel electrolyte composed of LiClO_4_ in a poly(vinylidene fluoride‐co‐hexafluoropropylene) (PVDF‐HFP) membrane, the authors demonstrated various batteries with transmittances of 78%, 60%, and 30% and corresponding energy densities of 5, 10, and 20 Wh L^−1^.^[^ ^193^ ^]^ For supercapacitors applications, the use of mesh‐like, orderly patterned, and interdigital films enabled light transmission through porous designs, allowing electrodes with larger thicknesses with little effect on the transparency.^[^ ^194^ ^]^ Another work shown in Figure 5b takes advantage of the multifunctional properties of transparent InGaZnO to build a transparent and monolithically integrated microsystem.^[^ ^156^ ^]^ In this work, transparent InGaZnO serves as the anode of the battery, as the channel of the thin‐film transistor, and as the photosensitive layer of the photodetector, with the motivation of eased fabrication process, transparency through the whole stack, and reduced energy loss between layers. Energy harvesting devices such as triboelectric nanogenerators,^[^ ^130^, ^155^, ^195^, ^196^, ^197^ ^]^ and photovoltaic devices^[^ ^198^, ^199^, ^200^ ^]^ ideally convert external stimuli into electrical signals, eliminating the need to charge or replace an energy storage device. The balance between transparency and efficiency of photovoltaic devices was studied trough two parameters: thickness of the photovoltaic film and donor–acceptor ratio.^[^ ^198^ ^]^ The authors report that the thickness of the photovoltaic film determines the device's transparency; while, affecting its efficiency, whereas the donor–acceptor ratio mainly impacts the efficiency, with little effect on transparency. By balancing these two parameters, they achieve efficiency as high as 4.06% and 2.38% with corresponding average visible transmission over 70% and 80%, respectively.

## 5 Concealing Approach to Realize Invisibility

We have discussed the approaches to render the electronics invisible by assembling transparent materials. The drawback of this strategy is that the efforts put on the transparency aspect often come at the expense of the materials’ electrical properties. This part presents research works based on non‐transparent materials that use different concealing strategies to make the devices unperceivable to others. Those strategies imply coloring the device to conceal it, hiding the electronics into our everyday accessories, or seamlessly integrating it into clothes.

### 5.1 Integrating to Normal Fashion

Electronic textiles were widely explored to conceal and integrate electronics into our everyday life. Clothes present the advantages to be used universally, and their large coverage of the body allows a great number of placement choices according to the functionalities. Applications range from remote healthcare, smart health and IoT, to safety industry, smart fashion, and the automobile industry. To meet clothes’ softness and comfort, integrated devices should be soft and stretchable and able to withstand a high level of repetitive deformations.

**Figure 6:**
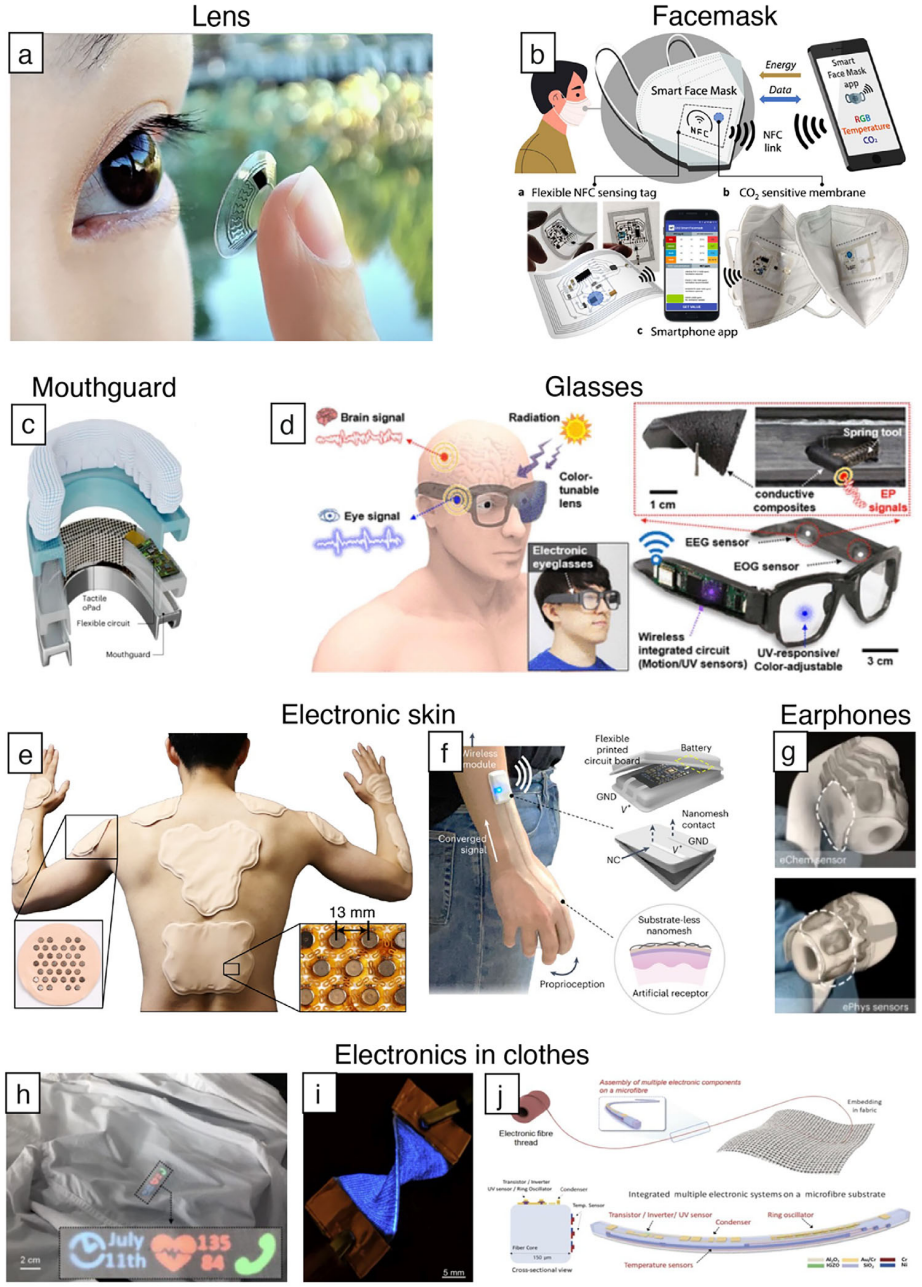
Strategies to conceal electronics in everyday clothes and accessories. a) Photos of a smart contact lens with the dual function of detecting pressure and temperature. Reproduced with permission.^[^ ^201^ ^]^ Copyright 2024, Springer Nature. b) Smart facial mask for CO_2_ detection. Reproduced with permission.^[^ ^202^ ^]^ Copyright 2022, Springer Nature. c) Tactile oral pad capturing teeth occlusal and tongue sliding action for machine control application. Reproduced with permission.^[^ ^203^ ^]^ Copyright 2024, Springer Nature. d) Smart glasses for real‐time sensing of multiple parameters (brain, eye, movement, and radiation signals). Reproduced with permission.^[^ ^204^ ^]^ Copyright 2020, American Chemical Society. Electronic skins: e) Haptic interfaces placed on skin with various designs fitting the anatomical structures of the body. Reproduced with permission.^[^ ^205^ ^]^ Copyright 2022, Springer Nature; f) Nanomesh cutaneous receptors connected to a wireless Bluetooth module for hand task recognition. Reproduced with permission.^[^ ^206^ ^]^ Copyright 2022, Springer Nature. g) Lactate in sweat and brain activity sensors integrated on earphones. Reproduced with permission.^[^ ^207^ ^]^ Copyright 2023, Springer Nature. In clothes: h) textile‐OLEDs display. Reproduced with permission.^[^ ^208^ ^]^ Copyright 2020, Springer Nature; i) light‐emitting display sandwiched between textile layers. Reproduced with permission.^[^ ^209^ ^]^ Copyright 2020, Elsevier; and j) e‐textile integrating multifunctional electronic fibers. Reproduced with permission.^[^ ^210^ ^]^ Copyright 2022, Springer Nature.

Applications such as displays benefit from being integrated into a soft and wearable form. Compared to rigid smart watches with sizes limited to reduce discomfort, clothes offer a larger surface area to display information. Different strategies were developed to integrate displays in clothes. One method consisted in first fabricating a stretchable display that would be later interfaced with the textile platform to realize a final textile‐based display.^[^ ^208^, ^209^, ^211^ ^]^ **Figure** **6**h shows a display made of a thin film of organic light‐emitting diodes (OLEDs) that is attached to a strain buffer layer before being fixed to textile in a pressure‐controlled process.^[^ ^208^ ^]^ This results in a stress‐lowering structured flexible platform, allowing the textile to be bent and stretched with limited transfer of deformations to the display. A textile‐like display is obtained by sandwiching a layer of electroluminescent Ecoflex between two layers of gold‐coated stretchable woven textiles.^[^ ^209^ ^]^ The result is a stretchable electroluminescent hybrid display with a textile surface feeling, as shown in Figure 6i. Another strategy consists in integrating functional threads in a twisted fiber structure, before weaving them, in the same manner as a textile would be fabricated.^[^ ^43^, ^212^, ^213^ ^]^ With this method, different functionalities can be integrated in textile, such as energy‐supplying module, sensing module, and display module. Especially, researchers have investigated ways to integrate computational electronics into textiles to build more advanced and complete smart systems. One strategy is the fabrication of an electronic microfiber integrating various components such as transistors, inverters, ring oscillators, and thermocouples, with precise micro‐patterning of semiconductor and electrode.^[^ ^210^ ^]^ The microfiber can then be woven into standard fabrics to functionalize it, as schematized in Figure 6j. This approach is promising as it results in a high density of computational electronics. To power wearable systems, energy harvesting devices are developed based on thermoelectric fibers integrated into textiles.^[^ ^43^, ^214^ ^]^ The thermoelectric threads are composed of alternately doped carbon nanotube fibers wrapped with acrylic fibers^[^ ^43^ ^]^ or gelatin‐extruded fibers.^[^ ^214^ ^]^ The textile generator can harness energy from the body heat and achieve a peak power density of 70 mW m⁻^2^ at a temperature difference of 44 K.

### 5.2 Blending in into Accessories

Other research works have explored concealing devices into everyday accessories, such as smart glasses,^[^ ^164^, ^204^, ^215^ ^]^ earphones,^[^ ^207^, ^216^ ^]^ facemask,^[^ ^202^, ^217^, ^218^, ^219^ ^]^ or even in mouth pads.^[^ ^203^, ^220^ ^]^

Due to the worldwide infection by SARS‐CoV‐2, many research works have focused on ways to make self‐diagnosing more accessible to the public for faster and more efficient treatment. The generalization of facemasks around the world made them logical platform to integrate these technologies. By integrating Near‐Field Communication (NFC) antennas, different wireless and battery‐free breath tests were developed for infection and symptoms severity diagnosis of SARS‐CoV‐2,^[^ ^219^ ^]^ or CO_2_ monitoring,^[^ ^202^ ^]^ as the system shown in Figure 6b. With the objective of popularizing and easing self‐tests indoors and outdoors, they demonstrated that their technology could be integrated into wristband, inside facemask, in a small carriable form or even on a necklace.

Other exploited accessories are glasses. Figure 6d shows 3D‐printed glasses integrating various physiological and environmental sensors, such as EEG, EOG, acceleration, and UV sensors.^[^ ^204^ ^]^ Motivation is human–machine interface based on eye‐tracking system integrated into the glasses,^[^ ^215^ ^]^ displaying sensitive data that are only visible to the user. Another location to dissimulate electronics is on earphones. Figure 6g shows specific sensors for the monitoring of brain activity and lactate in sweat,^[^ ^207^ ^]^ strategically placed in the ear without it being obtrusive to other people. Another application is EEG for drowsiness monitoring for drivers and pilots.^[^ ^216^ ^]^ Functionalizing oral pads have been explored for various applications. Figure 6c shows a control device developed for tetraplegic people, with the smart mouthguard being used for typing, gaming, and navigating a wheelchair through tongue sliding and teeth clicking.^[^ ^203^ ^]^ Other applications in medical care can also be concealed inside the mouth for accurate and early detection of oral diseases such as dental caries.^[^ ^220^ ^]^ A fluorescent mouthguard consisting of highly sensitive zinc oxide‐poly(dimethylsiloxane) (ZnO‐PDMS) nanocomposite detects volatile sulfur compounds secreted in oral cavities. They demonstrate the successful uncovering of precise locations of hidden dental lesion sites. Figure 6f shows a hand tasks recognition device that relies on nanomesh receptor coupled with an unsupervised meta‐learning framework for efficient, user‐independent recognition of hand tasks.^[^ ^206^ ^]^ The sensors can be attached to the surface of the hand and function with printed circuit boards encapsulated into a soft packaging, that should be attached to the forearm and hidden inside the sleeve.

### 5.3 Camouflage On‐Skin

Various applications such as haptic interfaces, sweat analysis, or hand movement‐based machine controllers require the electronic to be in close contact with skin. Haptic interfaces can be used to add sensations of touch to create augmented reality experiences, for applications in sports, medical care, orthopedic rehabilitation, entertainment systems, and so on. Haptic interfaces are devices with dimensions that are typically affected by the skin natural tactile resolution or the two‐point discrimination threshold for mechanical sensation. Some applications, in augmented reality games for example, need to apply tactile feedback on different parts of the body. For these reasons, haptic systems often require large surface areas. They also need to be integrated very closely to the skin to properly function; having them integrated inside clothes is not sufficient in terms of contact. Researchers have developed an electronic skin embedding vibrating motors to stimulate skin mechanoreceptors on a large area, shown in Figure 6e.^[^ ^205^ ^]^ The large area device is concealed with a skin‐color silicone encapsulating layer, and specifically designed forms allow close fitting to the body. With a similar but more advanced focus on concealing, researchers have developed an approach combining prosthetic makeup called “Morphace” with transformative wearables that generate dynamic output modalities such as sweating, tearing, or on‐demand patterns such as rosy cheeks or freckles.^[^ ^221^ ^]^ Other approaches include hiding electronics such as thin sweat analysis sensors under entertaining tattoo coverage.^[^ ^137^ ^]^ Electrotherapy devices for the healing of chronic wounds were also developed as ultra‐thin electronic layers that could be concealed under a skin‐dressing or bandage.^[^ ^222^ ^]^

### 5.4 Ultraminiaturize

Miniaturization of silicon‐based integrated circuits has majorly contributed to the shrinking of electronic devices and their integration into wearable designs. When the system is complex with several elements interacting in a limited space, miniaturized conventional materials with state‐of‐the‐art electronic performances can be favored over emerging transparent materials. Smart lenses for theragnostic (portmanteau of therapeutic and diagnostic) devices are a good example of ultraminiaturized devices.^[^ ^201^, ^223^, ^224^, ^225^, ^226^ ^]^ One of their main applications is the monitoring of glaucoma, the leading cause of irreversible blindness worldwide, requiring continuous surveillance of the intraocular pressure (IOP) and immediate treatment adjustment. An intelligent wireless contact lens for both in situ electrical sensing of IOP and anti‐glaucoma drug delivery was developed.^[^ ^225^ ^]^ The lens comprises an IOP ultra‐sensitive cantiveler, which can trigger iontophotoresis electrodes to allow stored anti‐glaucoma drugs to be delivered to the surface of the eye. Another simple yet effective approach was developed with conductive Ag/SEBS, dielectric Silbione, and PDMS inks printed in a series resistor‐inductor‐capacitor (RLC) resonant circuit.^[^ ^227^ ^]^ With a single 200‐µm wide line, the circuit is barely noticeable. The RLC can then be transferred onto commercialized lenses without altering their medical characteristics and can be continuously worn for 24 h. The changes in IOP induce a change in the highly compressible Silbione capacitor; and therefore, in the RLC resonance, which can be measured through an antenna integrated on glasses or a sleeping mask throughout the whole day.

## 6 Conclusion and Future Prospects

In this review, we summarize recent advances in unperceivable electronics, ranging from intrinsically transparent materials to concealment strategies and device applications. Market studies and user‐experience feedback have shown that the consumer acceptance is greatly influenced by the discretion of the device. Unperceivable technologies represent an innovative direction for future wearable technologies. Their invisibility ensures that the devices can be worn without attracting attention, significantly reducing psychological pressure on the user. Wearers can have their physiological status monitored continuously, even in social settings; and while, interacting with others. Moreover, unperceivable electronics offer improved comfort and more accurate data collection, thanks to their form factor that perfectly conforms to the body.

Despite significant advancements in unperceivable electronics, several technological challenges remain before they reach their full potential, enter the consumer market, and be applied in real‐world scenarios. One current limitation is the power supplying device. Various wearable batteries with soft or stretchable properties were developed, but their energy density remains far below the performance level of conventional rigid batteries. Further development of energy harvesting technologies such as tribogenerators will enable device operation for extended periods of time without requiring frequent charging or replacing of a battery. Another technical challenge to address is the need for durable material with environmental adaptability. When directly worn on skin, unperceivable electronics must withstand long‐term daily use, including friction from clothes and exposure to humidity during daily activities such as bathing and exercise. Heat management will also impact the lifespan, accuracy, and performances of electronic devices. Poor heat dissipation leading to overheating can affect the measurements consistency of devices embedding sensors. Final assembly of the various elements composing the electronic system including the sensors, processors, energy devices, and communication modules, also remains a significant challenge. Coordinating these elements requires specifically developed interconnecting and communication methods, which have not yet been extensively studied. Future research may partially rely on nanotechnologies to miniaturize components such as power sources and computing chips, to achieve a complete system with reduced visibility.

Apart from technical challenges, societal and regulatory issues arise with the market introduction of unperceivable wearable technologies. The proliferation of wearable devices that collect, store, and transmit large amounts of personal information inevitably brings attention to data theft risks. Users of wearable technologies are also concerned about their data being shared with third parties without their informed and unambiguous consent.^[^ ^228^ ^]^ From a non‐user perspective, the market introduction of invisible, portable new technologies may also raise concerns about privacy and consent. In the near future, we can expect important changes in legal standards in response to the rapid growth of these new technologies.

Although unperceivable wearables technologies still face many technological and societal challenges, their applications are very promising. Advanced healthcare systems integrating invisible electrodes on the face could provide early detection of neurological diseases such as Parkinson's, Alzheimer's, and epilepsy, enabling timely medical intervention and potentially improved patient outcomes. For human–machine interfaces, unperceivable sensors placed on‐skin enable smooth and intuitive control methods, such as steering the flight direction of a quadcopter using eyeball movement.^[^ ^79^ ^]^ Applications in human capability augmentation and biomedical devices are also highly desired, such as controlling a motorized wheelchair with a functionalized mouthguard for an individual with physical impairments.^[^ ^203^ ^]^ In the Internet‐of‐Things applications, smart clothing and smart homes are seamlessly integrated to enable intelligent control and data collection, enhancing the quality of life.

## Conflict of Interest

The authors declare no conflict of interest.

## References

[1] H. Hinrichs , M. Scholz , A. K. Baum , J. W. Y. Kam , R. T. Knight , H.‐J. Heinze , Sci. Rep. 2020, 10, 5218. 32251333 10.1038/s41598-020-62154-0 PMC7090045

[2] P. A. Constable , M. Bach , L. J. Frishman , B. G. Jeffrey , A. G. Robson , Doc. Ophthalmol. 2017, 134, 1. 28110380 10.1007/s10633-017-9573-2 PMC5309273

[3] J. Reeves , C. Starbuck , C. Nester , J. Electromyogr. Kinesiol. 2020, 54, 102461. 32905962 10.1016/j.jelekin.2020.102461

[4] S. R. Soekadar , M. Witkowski , N. Vitiello , N. Birbaumer , Biomed. Tech. Berl. 2015, 60, 199. 25490027 10.1515/bmt-2014-0126

[5] L. Inzelberg , D. Rand , S. Steinberg , M. David‐Pur , Y. Hanein , Sci. Rep. 2018, 8, 2058. 29391503 10.1038/s41598-018-20567-y PMC5794977

[6] Y. Wang , S. Lee , T. Yokota , H. Wang , Z. Jiang , J. Wang , M. Koizumi , T. Someya , Sci. Adv. 2020, 6, abb7043. 10.1126/sciadv.abb7043 PMC7423357 32851182

[7] J. Kim , J. R. Sempionatto , S. Imani , M. C. Hartel , A. Barfidokht , G. Tang , A. S. Campbell , P. P. Mercier , J. Wang , Adv. Sci. 2018, 5, 1800880. 10.1002/advs.201800880 PMC6193173 30356971

[8] Q. Zhou , C. Zhu , H. Xue , L. Jiang , J. Wu , ACS Appl. Mater. Interfaces 2024, 16, 35268. 38916408 10.1021/acsami.4c03143

[9] T. Shimura , S. Sato , P. Zalar , N. Matsuhisa , Adv. Electron. Mater. 2022, 9, 2200512.

[10] M. Wang , Y. Yang , J. Min , Y. Song , J. Tu , D. Mukasa , C. Ye , C. Xu , N. Heflin , J. S. McCune , T. K. Hsiai , Z. Li , W. Gao , Nat. Biomed. Eng. 2022, 6, 1225. 35970928 10.1038/s41551-022-00916-z PMC10432133

[11] S. Yoon , J. K. Sim , Y.‐H. Cho , Sci. Rep. 2016, 6, 23468. 27004608 10.1038/srep23468 PMC4804278

[12] Y. Jin , J. T. Alvarez , E. L. Suitor , K. Swaminathan , A. Chin , U. S. Civici , R. W. Nuckols , R. D. Howe , C. J. Walsh , Nat. Commun. 2024, 15, 5756. 38982087 10.1038/s41467-024-50038-0 PMC11233567

[13] S. Nakata , M. Shiomi , Y. Fujita , T. Arie , S. Akita , K. Takei , Nat. Electron. 2018, 1, 596.

[14] J. H. Shin , J. Kwon , J. U. Kim , H. Ryu , J. Ok , S. Joon Kwon , H. Park , T.‐I. Kim , npj Flexible Electron. 2022, 6, 32.

[15] A. Moin , A. Zhou , A. Rahimi , A. Menon , S. Benatti , G. Alexandrov , S. Tamakloe , J. Ting , N. Yamamoto , Y. Khan , F. Burghardt , L. Benini , A. C. Arias , J. M. Rabaey , Nat. Electron. 2020, 4, 54.

[16] Y. Li , G. Matsumura , Y. Xuan , S. Honda , K. Takei , Adv. Funct. Mater. 2024, 34, 2313824.

[17] S. Liu , Y. Wu , L. Jiang , W. Xie , B. Davis , M. Wang , L. Zhang , Y. Liu , S. Xing , M. D. Dickey , W. Bai , ACS Appl. Mater. Interfaces 2024, 16, 46538. 39087831 10.1021/acsami.4c10539 PMC12961969

[18] J. Kim , H. J. Shim , J. Yang , M. K. Choi , D. C. Kim , J. Kim , T. Hyeon , D.‐H. Kim , Adv. Mater. 2017, 29, 1700217. 10.1002/adma.201700217 28833644

[19] J. H. Koo , D. C. Kim , H. J. Shim , T.‐H. Kim , D.‐H. Kim , Adv. Funct. Mater. 2018, 28, 1801834.

[20] J. H. Koo , S. Jeong , H. J. Shim , D. Son , J. Kim , D. C. Kim , S. Choi , J.‐I. Hong , D.‐H. Kim , ACS Nano 2017, 11, 10032. 28837773 10.1021/acsnano.7b04292

[21] H. E. Lee , J. H. Shin , J. H. Park , S. K. Hong , S. H. Park , S. H. Lee , J. H. Lee , I.‐S. Kang , K. J. Lee , Adv. Funct. Mater. 2019, 29, 1808075.

[22] L. Miao , S. Zhu , C. Liu , J. Gao , Z. Zhang , Y. Peng , J.‐L. Chen , Y. Gao , J. Liang , T. Mori , Nat. Commun. 2024, 15, 8516. 39353932 10.1038/s41467-024-52841-1 PMC11445405

[23] D. Lv , Q. Jiang , Y. Shang , D. Liu , Npj Flexible Electron. 2022, 6, 38.

[24] J. Min , S. Demchyshyn , J. R. Sempionatto , Y. Song , B. Hailegnaw , C. Xu , Y. Yang , S. Solomon , C. Putz , L. E. Lehner , J. F. Schwarz , C. Schwarzinger , M. C. Scharber , E. Shirzaei Sani , M. Kaltenbrunner , W. Gao , Nat. Electron. 2023, 6, 630 38465017 10.1038/s41928-023-00996-y PMC10923186

[25] C. Peng , N. Xi , Z. Hong , J. Hamari , Proceedings of the Annual Hawaii International Conference on System Sciences, 2022, pp. 1–10. 10.24251/hicss.2022.505 PMC9076271 35528964

[26] Z. Yin , J. Yan , S. Fang , D. Wang , D. Han , Ann. Transl. Med. 2022, 10, 629. 35813345 10.21037/atm-21-5510 PMC9263785

[27] G. C. S. Schwambach , Ó. H. López , M. K. Sott , L. P. Carvalho Tedesco , R. F. Molz , Technol. Soc. 2022, 68, 101840.

[28] C.‐C. Chen , H.‐S. Shih , International Journal of the Analytic Hierarchy Process, Creative Decisions Foundation 2014.

[29] A. Fitzpatrick , Why Google Glass Isn't the Future, https://time.com/3588143/google‐glass/ (accessed: February 2025).

[30] D. Y. Meier , P. Barthelmess , W. Sun , F. Liberatore , J. Med. Internet Res. 2020, 22, 18801. 10.2196/18801 PMC7644382 33090108

[31] T. Pangarkar , Wearable Technology Statistics 2025 By Tech and Human, https://scoop.market.us/wearable‐technology‐statistics/ (accessed: February 2025)

[32] E. L. Mahoney , D. F. Mahoney , Am. J. Alzheimer's. Dis. Other Dement. 2010, 25, 527. 10.1177/1533317510376944 PMC10845356 20702501

[33] J. Jang , J. Kim , H. Shin , Y.‐G. Park , B. J. Joo , H. Seo , J.‐E. Won , D. W. Kim , C. Y. Lee , H. K. Kim , J.‐U. Park , Sci. Adv. 2021, 7, abf7194. 10.1126/sciadv.abf7194 PMC8011975 33789904

[34] G. Ghosh , M. Meeseepong , A. Bag , A. Hanif , M. V. Chinnamani , M. Beigtan , Y. Kim , N.‐E. Lee , Mater. Today 2022, 57, 43.

[35] L. Shi , Z. Li , M. Chen , Y. Qin , Y. Jiang , L. Wu , Nat. Commun. 2020, 11, 3529. 32669556 10.1038/s41467-020-17298-y PMC7363923

[36] I. Wicaksono , C. I. Tucker , T. Sun , C. A. Guerrero , C. Liu , W. M. Woo , E. J. Pence , C. Dagdeviren , npj Flexible Electron. 2020, 4, 5. 10.1038/s41528-020-0068-y PMC7222957 38624354

[37] D. Won , J. Bang , S. H. Choi , K. R. Pyun , S. Jeong , Y. Lee , S. H. Ko , Chem. Rev. 2023, 123, 9982. 37542724 10.1021/acs.chemrev.3c00139 PMC10452793

[38] K. Kim , Y.‐G. Park , B. G. Hyun , M. Choi , J.‐U. Park , Adv. Mater. 2019, 31, 1804690. 10.1002/adma.201804690 30556173

[39] Y.‐W. Lim , J. Jin , B.‐S. Bae , Adv. Mater. 2020, 32, 1907143. 10.1002/adma.201907143 32187405

[40] A. Tricoli , N. Nasiri , S. De , Adv. Funct. Mater. 2017, 27, 1605271.

[41] M. Bian , Y. Qian , H. Cao , T. Huang , Z. Ren , X. Dai , S. Zhang , Y. Qiu , R. Si , L. Yang , S. Yin , ACS Appl. Mater. Interfaces 2023, 15, 13307. 36880523 10.1021/acsami.2c21996

[42] H. Xu , J. Liu , J. Zhang , G. Zhou , N. Luo , N. Zhao , Adv. Mater. 2017, 29, 1700975. 10.1002/adma.201700975 28612929

[43] T. Sun , B. Zhou , Q. Zheng , L. Wang , W. Jiang , G. J. Snyder , Nat. Commun. 2020, 11, 572. 31996675 10.1038/s41467-020-14399-6 PMC6989526

[44] Z. Zhang , C. Du , H. Jiao , M. Zhang , Adv. Electron. Mater. 2020, 6, 1901133.

[45] L. Snarski , I. Biran , T. Bendikov , I. Pinkas , M. A. Iron , I. Kaplan‐Ashiri , H. Weissman , B. Rybtchinski , Adv. Funct. Mater. 2024, 34, 2309742.

[46] S. Kabiri Ameri , R. Ho , H. Jang , L. Tao , Y. Wang , L. Wang , D. M. Schnyer , D. Akinwande , N. Lu , ACS Nano 2017, 11, 7634. 28719739 10.1021/acsnano.7b02182

[47] D. Song , X. Li , M. Jang , Y. Lee , Y. Zhai , W. Hu , H. Yan , S. Zhang , L. Chen , C. Lu , K. Kim , N. Liu , Adv. Mater. 2023, 35, 2304956. 10.1002/adma.202304956 37533340

[48] Y. Jiang , Z. Zhang , Y.‐X. Wang , D. Li , C.‐T. Coen , E. Hwaun , G. Chen , H.‐C. Wu , D. Zhong , S. Niu , W. Wang , A. Saberi , J.‐C. Lai , Y. Wu , Y. Wang , A. A. Trotsyuk , K. Y. Loh , C.‐C. Shih , W. Xu , K. Liang , K. Zhang , Y. Bai , G. Gurusankar , W. Hu , W. Jia , Z. Cheng , R. H. Dauskardt , G. C. Gurtner , J. B.‐H. Tok , K. Deisseroth , et al., Science 2022, 375, 1411. 35324282 10.1126/science.abj7564

[49] S. Cheng , Z. Lou , L. Zhang , H. Guo , Z. Wang , C. Guo , K. Fukuda , S. Ma , G. Wang , T. Someya , H.‐M. Cheng , X. Xu , Adv. Mater. 2023, 35, 2206793. 10.1002/adma.202206793 36267034

[50] Y. Zhu , Y. Deng , P. Yi , L. Peng , X. Lai , Z. Lin , Adv. Mater. Technol. 2019, 4, 1900413.

[51] X. Chen , G. Xu , G. Zeng , H. Gu , H. Chen , H. Xu , H. Yao , Y. Li , J. Hou , Y. Li , Adv. Mater. 2020, 32, 1908478. 10.1002/adma.201908478 32103580

[52] Y.‐M. Ju , J.‐W. Park , Y.‐R. Jang , S. S. Park , H.‐S. Kim , Int. J. Precis. Eng. Manuf. ‐ Green Technol. 2023, 11, 203.

[53] B. P. Yalagala , S. A. Sankaranarayanan , A. K. Rengan , S. R. K. Vanjari , ACS Sustainable Chem. Eng. 2022, 10, 4473.

[54] S. Cho , S. Kang , A. Pandya , R. Shanker , Z. Khan , Y. Lee , J. Park , S. L. Craig , H. Ko , ACS Nano 2017, 11, 4346. 28397485 10.1021/acsnano.7b01714

[55] S. Kang , S. Cho , R. Shanker , H. Lee , J. Park , D.‐S. Um , Y. Lee , H. Ko , Sci. Adv. 2018, 4, aas8772. 10.1126/sciadv.aas8772 PMC6070362 30083604

[56] C. Qin , Q. Sun , Y. Chen , S. Fahad , J. Wu , Y. Dong , H. Yu , M. Wang , npj Flexible Electron. 2024, 8, 26.

[57] F. Xu , Y. Zhu , Adv. Mater. 2012, 24, 5117. 22786752 10.1002/adma.201201886

[58] P. Won , J. J. Park , T. Lee , I. Ha , S. Han , M. Choi , J. Lee , S. Hong , K.‐J. Cho , S. H. Ko , Nano Lett. 2019, 19, 6087. 31411037 10.1021/acs.nanolett.9b02014

[59] Y. Dai , K. Qi , K. Ou , Y. Song , Y. Zhou , M. Zhou , H. Song , J. He , H. Wang , R. Wang , ACS Appl. Mater. Interfaces 2023, 15, 11244. 36791272 10.1021/acsami.2c20322

[60] G. Higueros , K. Wang , C. Sui , P.‐C. Hsu , ACS Nano 2024, 18, 13808. 38747521 10.1021/acsnano.4c02093

[61] J. J. Kim , K. Shuji , J. Zheng , X. He , A. Sajjad , H. Zhang , H. Su , W. C. H. Choy , Nat. Commun. 2024, 15, 2070. 38453936 10.1038/s41467-024-46243-6 PMC10920808

[62] H. Meskher , T. Ragdi , A. K. Thakur , S. Ha , I. Khelfaoui , R. Sathyamurthy , S. W. Sharshir , A. K. Pandey , R. Saidur , P. Singh , F. Sharifian Jazi , I. Lynch , Crit. Rev. Anal. Chem. 2024, 54, 2398. 36724894 10.1080/10408347.2023.2171277

[63] W. Zhang , J. Miao , X. Zuo , X. Zhang , L. Qu , J. Mater. Chem. C 2022, 10, 14027.

[64] X. Cheng , Z. Pan , C. Fan , Z. Wu , L. Ding , L.‐M. Peng , Sci. Adv. 2024, 10, adl1636. 10.1126/sciadv.adl1636 PMC10959407 38517964

[65] B. Liang , Z. Zhang , W. Chen , D. Lu , L. Yang , R. Yang , H. Zhu , Z. Tang , X. Gui , Nanomicro Lett. 2019, 11, 92. 34138033 10.1007/s40820-019-0323-8 PMC7770666

[66] P. Liu , E.‐X. Ding , Z. Xu , X. Cui , M. Du , W. Zeng , A. Karakassides , J. Zhang , Q. Zhang , F. Ahmed , H. Jiang , P. Hakonen , H. Lipsanen , Z. Sun , E. I. Kauppinen , ACS Nano 2024, 18, 18900. 38997111 10.1021/acsnano.4c01087 PMC11271656

[67] Z. Xu , D. Fox , N. Wei , P. Liu , Y. Wen , R. Gao , A. R. Harutyunyan , H. Jiang , Q. Zhang , E. I. Kauppinen , Nano Today 2025, 61, 102635.

[68] M. M. Hossain , B. M. Li , B. Sennik , J. S. Jur , P. D. Bradford , npj Flexible Electron. 2022, 6, 97.

[69] F. Chen , Y. Huang , R. Li , S. Zhang , Q. Jiang , Y. Luo , B. Wang , W. Zhang , X. Wu , F. Wang , P. Lyu , S. Zhao , W. Xu , F. Wei , R. Zhang , Sci. Adv. 2022, 8, abn5882. 10.1126/sciadv.abn5882 PMC9242455 35767610

[70] K. Kawasaki , I. Harada , K. Akaike , Q. Wei , Y. Koshiba , S. Horike , K. Ishida , Commun. Mater. 2024, 5, 21.

[71] Y. Wang , T. Wang , L. Xiang , R. Huang , G. Long , W. Wang , M. Xi , J. Tian , W. Li , X. Deng , Q. Gong , T. Bai , Y. Chen , H. Liu , Y. Xia , X. Liang , Q. Chen , L.‐M. Peng , Y. Hu , Sci. Adv. 2024, 10, adq6022. 10.1126/sciadv.adq6022 PMC11378910 39241060

[72] W. Sun , J. Shen , Z. Zhao , N. Arellano , C. Rettner , J. Tang , T. Cao , Z. Zhou , T. Ta , J. K. Streit , J. A. Fagan , T. Schaus , M. Zheng , S.‐J. Han , W. M. Shih , H. T. Maune , P. Yin , Science 2020, 368, 874. 32439790 10.1126/science.aaz7440

[73] K. S. Novoselov , A. K. Geim , S. V. Morozov , D. Jiang , Y. Zhang , S. V. Dubonos , I. V. Grigorieva , A. A. Firsov , Science 2004, 306, 666. 15499015 10.1126/science.1102896

[74] L. Meng , W. Wang , B. Xu , J. Qin , K. Zhang , H. Liu , ACS Nano 2023, 17, 4180. 36826227 10.1021/acsnano.2c10999

[75] Y. S. Woo , Micromachines 2018, 10, 13.30587828

[76] M. Zhang , H. Yang , H. Li , L. Tong , C. Su , K. Feng , Q. Wang , H. Yan , S. Yin , J. Alloys Compd. 2023, 967, 171724.

[77] Y. Zhu , M. C. Hartel , N. Yu , P. R. Garrido , S. Kim , J. Lee , P. Bandaru , S. Guan , H. Lin , S. Emaminejad , N. R. de Barros , S. Ahadian , H.‐J. Kim , W. Sun , V. Jucaud , M. R. Dokmeci , P. S. Weiss , R. Yan , A. Khademhosseini , Small Methods 2022, 6, 2100900. 10.1002/smtd.202100900 PMC8852346 35041280

[78] C. Huang , Z. Hao , Z. Wang , H. Wang , X. Zhao , Y. Pan , Adv. Mater. Technol. 2022, 7, 2101131.

[79] S. K. Ameri , M. Kim , I. A. Kuang , W. K. Perera , M. Alshiekh , H. Jeong , U. Topcu , D. Akinwande , N. Lu , npj 2D Mater. Appl. 2018, 2, 19.

[80] A. Yildirim , J. C. Grant , G. Song , S. Yook , Z. Mutlu , S. Peana , A. Dhanabal , S. K. Sinha , R. Daniels , K. M. Bellisario , G. A. Sotzing , D. H. Huston , B. C. Pijanowksi , R. Rahimi , Y. Liu , M. Cakmak , Adv. Mater. Technol. 2020, 5, 2000296.

[81] K. S. Novoselov , A. Mishchenko , A. Carvalho , A. H. Castro Neto , Science 2016, 353, aac9439. 27471306 10.1126/science.aac9439

[82] A. VahidMohammadi , J. Rosen , Y. Gogotsi , Science 2021, 372, abf1581. 10.1126/science.abf1581 34112665

[83] Y. Bai , K. Zhou , N. Srikanth , J. H. L. Pang , X. He , R. Wang , RSC Adv. 2016, 6, 35731.

[84] N. Li , J. Huo , Y. Zhang , B. Ye , X. Chen , X. Li , S. Xu , J. He , X. Chen , Y. Tang , Y. Zhu , K. Ling , R. Zhu , Sep. Purif. Technol. 2024, 330, 125325.

[85] W. Jiang , S. Lee , K. Zhao , K. Lee , H. Han , J. Oh , H. Lee , H. Kim , C. M. Koo , C. Park , ACS Nano 2022, 16, 9203. 35588151 10.1021/acsnano.2c01514

[86] Q. Fan , J. Miao , X. Liu , X. Zuo , W. Zhang , M. Tian , S. Zhu , L. Qu , X. Zhang , Nano Lett. 2022, 22, 740. 35019663 10.1021/acs.nanolett.1c04185

[87] Z. Duan , M. Yuan , Z. Liu , W. Pei , K. Jiang , L. Li , G. Shen , Small 2024, 20, 2309785. 10.1002/smll.202309785 38377279

[88] X. Jin , X. Guo , J. Liu , Q. Guo , B. Lei , J. Wang , Cell Rep. Phys. Sci. 2023, 4, 101286.

[89] C. Ma , Q. Yuan , H. Du , M.‐G. Ma , C. Si , P. Wan , ACS Appl. Mater. Interfaces 2020, 12, 34226. 32673490 10.1021/acsami.0c10750

[90] M. Shi , M. Shen , X. Guo , X. Jin , Y. Cao , Y. Yang , W. Wang , J. Wang , ACS Nano 2021, 15, 11396. 34165297 10.1021/acsnano.1c00903

[91] S. Lee , E. H. Kim , S. Yu , H. Kim , C. Park , S. W. Lee , H. Han , W. Jin , K. Lee , C. E. Lee , J. Jang , C. M. Koo , C. Park , ACS Nano 2021, 15, 8940. 33983015 10.1021/acsnano.1c01621

[92] Y. Li , Y. Pang , L. Wang , Q. Li , B. Liu , J. Li , S. Liu , Q. Zhao , Adv. Mater. 2024, 36, 2310973. 10.1002/adma.202310973 38185875

[93] T. Someya , Z. Bao , G. G. Malliaras , Nature 2016, 540, 379. 27974769 10.1038/nature21004

[94] M. Berggren , G. G. Malliaras , Science 2019, 364, 233. 31000650 10.1126/science.aaw9295

[95] Y. Wang , C. Zhu , R. Pfattner , H. Yan , L. Jin , S. Chen , F. Molina‐Lopez , F. Lissel , J. Liu , N. I. Rabiah , Z. Chen , J. W. Chung , C. Linder , M. F. Toney , B. Murmann , Z. Bao , Sci. Adv. 2017, 3, 1602076. 10.1126/sciadv.1602076 PMC5345924 28345040

[96] T. Shimura , S. Sato , T. Tominaga , S. Abe , K. Yamashita , M. Ashizawa , T. Kato , H. Ishikuro , N. Matsuhisa , Adv. Mater. Technol. 2023, 8, 2201992.

[97] M. Clevenger , H. Kim , H. W. Song , K. No , S. Lee , Sci. Adv. 2021, 7, abj8958. 10.1126/sciadv.abj8958 PMC8519566 34652946

[98] T. Ren , H. Yang , J. Zhang , K. Lv , D. Kong , F. Jiang , Y. Chang , P. Yu , J. Tao , D. Wang , N. Kong , Y. Shao , Adv. Eng. Mater. 2023, 25, 2301018.

[99] Y.‐Q. Zheng , Y. Liu , D. Zhong , S. Nikzad , S. Liu , Z. Yu , D. Liu , H.‐C. Wu , C. Zhu , J. Li , H. Tran , J. B.‐H. Tok , Z. Bao , Science 2021, 373, 88. 34210882 10.1126/science.abh3551

[100] D. Won , J. Kim , J. Choi , H. Kim , S. Han , I. Ha , J. Bang , K. K. Kim , Y. Lee , T.‐S. Kim , J.‐H. Park , C.‐Y. Kim , S. H. Ko , Sci. Adv. 2022, 8, abo3209. 10.1126/sciadv.abo3209 PMC9177068 35675404

[101] H. Fujita , M. Hao , S. Takeoka , Y. Miyahara , T. Goda , T. Fujie , Adv. Mater. Technol. 2022, 7, 2101486.

[102] Z. Zhu , G. Yang , R. Li , T. Pan , Microsyst. Nanoeng. 2017, 3, 17004. 31057859 10.1038/micronano.2017.4 PMC6445012

[103] H. Kamata , Y. Akagi , Y. Kayasuga‐Kariya , U.‐I. Chung , T. Sakai , Science 2014, 343, 873. 24558157 10.1126/science.1247811

[104] X. Cui , J. Li , Y. Hartanto , M. Durham , J. Tang , H. Zhang , G. Hooper , K. Lim , T. Woodfield , Adv. Healthcare Mater. 2020, 9, 1901648. 10.1002/adhm.201901648 32352649

[105] H. Wei , Z. Wang , H. Zhang , Y. Huang , Z. Wang , Y. Zhou , B. B. Xu , S. Halila , J. Chen , Chem. Mater. 2021, 33, 6731.

[106] H. Liu , D. Xu , B. Hu , J. Jiang , M. Li , D. Zhao , W. Zhai , J. Mater. Chem. A 2021, 9, 4692.

[107] Y. Go , H.‐Y. Park , Y. Zhu , K. Yoo , J. Kwak , S.‐H. Jin , J. Yoon , Adv. Funct. Mater. 2023, 33, 2215193.

[108] Y. Cai , J. Shen , C.‐W. Yang , Y. Wan , H.‐L. Tang , A. A. Aljarb , C. Chen , J.‐H. Fu , X. Wei , K.‐W. Huang , Y. Han , S. J. Jonas , X. Dong , V. Tung , Sci. Adv. 2020, 6, abb5367. 10.1126/sciadv.abb5367 PMC7695469 33246950

[109] J. Yu , M. Wang , C. Dang , C. Zhang , X. Feng , G. Chen , Z. Huang , H. Qi , H. Liu , J. Kang , J. Mater. Chem. C 2021, 9, 3635.

[110] X. Huang , Z. Zheng , H. Wang , W. Xu , M. Wu , M. Wang , C. Chen , L. Wan , R. Du , T. Zhu , Z. Huang , X. Wang , X. Wang , Q. Zhang , X. Jia , Adv. Funct. Mater. 2024, 34, 2312149.

[111] J. Luo , C. Sun , B. Chang , B. Zhang , K. Li , Y. Li , Q. Zhang , H. Wang , C. Hou , Adv. Funct. Mater. 2024, 34, 2400884.

[112] A. K. A. Aljarid , C. S. Boland , Adv. Funct. Mater. 2024, 34, 2405799.

[113] E. Kim , J.‐C. Lai , L. Michalek , W. Wang , C. Xu , H. Lyu , W. Yu , H. Park , Y. Tomo , S. E. Root , B. Lee , J. Park , B. Park , S. Wei , C. Zhao , Z. Bao , Adv. Funct. Mater. 2025, 35, 2411880.

[114] S. Huang , B. Zhang , Z. Shao , L. He , Q. Zhang , J. Jie , X. Zhang , Nano Lett. 2020, 20, 2478. 32142295 10.1021/acs.nanolett.9b05217

[115] S. Lee , S. Franklin , F. A. Hassani , T. Yokota , M. O. G. Nayeem , Y. Wang , R. Leib , G. Cheng , D. W. Franklin , T. Someya , Science 2020, 370, 966. 33214278 10.1126/science.abc9735

[116] Y. Wang , C. Zhao , J. Wang , X. Luo , L. Xie , S. Zhan , J. Kim , X. Wang , X. Liu , Y. Ying , Sci. Adv. 2021, 7, abe4553. 10.1126/sciadv.abe4553 PMC10964967 33523953

[117] Y. Isano , H. Fujita , K. Murakami , S. Ni , Y. Kurotaki , T. Takano , Y. Isoda , R. Matsuda , F. Nakamura , Y. Nishitai , N. Ochirkhuyag , K. Inoue , H. Kawakami , Y. Okubo , K. Ueno , T. Fujie , H. Ota , Adv. Mater. Technol. 2022, 7, 2200209.

[118] H. Souri , H. Banerjee , A. Jusufi , N. Radacsi , A. A. Stokes , I. Park , M. Sitti , M. Amjadi , Adv. Intell. Syst. 2020, 2, 2000039.

[119] Q. Yu , R. Ge , J. Wen , T. Du , J. Zhai , S. Liu , L. Wang , Y. Qin , Nat. Commun. 2022, 13, 778. 35140219 10.1038/s41467-022-28443-0 PMC8828782

[120] D. D. L. Chung , J. Mater. Sci. 2020, 55, 15367.

[121] L. Cai , L. Song , P. Luan , Q. Zhang , N. Zhang , Q. Gao , D. Zhao , X. Zhang , M. Tu , F. Yang , W. Zhou , Q. Fan , J. Luo , W. Zhou , P. M. Ajayan , S. Xie , Sci. Rep. 2013, 3, 3048. 24157842 10.1038/srep03048 PMC6505716

[122] D. J. Lipomi , M. Vosgueritchian , B. C.‐K. Tee , S. L. Hellstrom , J. A. Lee , C. H. Fox , Z. Bao , Nat. Nanotechnol. 2011, 6, 788. 22020121 10.1038/nnano.2011.184

[123] L. Xu , Z. Huang , Z. Deng , Z. Du , T. L. Sun , Z.‐H. Guo , K. Yue , Adv. Mater. 2021, 33, 2105306. 10.1002/adma.202105306 34647370

[124] M. Wang , Z. Yan , T. Wang , P. Cai , S. Gao , Y. Zeng , C. Wan , H. Wang , L. Pan , J. Yu , S. Pan , K. He , J. Lu , X. Chen , Nat. Electron. 2020, 3, 563.

[125] F. Yin , H. Lu , H. Pan , H. Ji , S. Pei , H. Liu , J. Huang , J. Gu , M. Li , J. Wei , Sci. Rep. 2019, 9, 2403. 30787401 10.1038/s41598-019-38931-x PMC6382792

[126] S. Lee , J. S. Kim , Y. Wang , Y. Tagawa , W. Wang , L. Sun , X. Liang , M. O. Goni Nayeem , T. Yokota , K. Fukuda , T. Someya , Device 2025, 3, 100559.

[127] K. Meng , X. Xiao , W. Wei , G. Chen , A. Nashalian , S. Shen , X. Xiao , J. Chen , Adv. Mater. 2022, 34, 2109357. 10.1002/adma.202109357 35044014

[128] K. L. Montero , M.‐M. Laurila , M. Peltokangas , M. Haapala , J. Verho , N. Oksala , A. Vehkaoja , M. Mäntysalo , ACS Appl. Electron. Mater. 2021, 3, 4362.

[129] Y. Zhang , Q. Lu , J. He , Z. Huo , R. Zhou , X. Han , M. Jia , C. Pan , Z. L. Wang , J. Zhai , Nat. Commun. 2023, 14, 1252. 36878931 10.1038/s41467-023-36885-3 PMC9988987

[130] J. He , Z. Xie , K. Yao , D. Li , Y. Liu , Z. Gao , W. Lu , L. Chang , X. Yu , Nano Energy 2021, 81, 105590.

[131] Y. Tang , P. Jin , Y. Wang , D. Li , Y. Chen , P. Ran , W. Fan , K. Liang , H. Ren , X. Xu , R. Wang , Y. M. Yang , B. Zhu , Nat. Commun. 2023, 14, 4961. 37587158 10.1038/s41467-023-40711-1 PMC10432415

[132] Q. Liu , Z. Liu , C. Li , K. Xie , P. Zhu , B. Shao , J. Zhang , J. Yang , J. Zhang , Q. Wang , C. F. Guo , Adv. Sci. 2020, 7, 2000348. 10.1002/advs.202000348 PMC7237840 32440489

[133] S. Chen , L. Sun , X. Zhou , Y. Guo , J. Song , S. Qian , Z. Liu , Q. Guan , E. M. Jeffries , W. Liu , Y. Wang , C. He , Z. You , Nat. Commun. 2020, 11, 1107. 32107380 10.1038/s41467-020-14446-2 PMC7046662

[134] Y. Song , J. Min , Y. Yu , H. Wang , Y. Yang , H. Zhang , W. Gao , Sci. Adv. 2020, 6, eaay9842. 32998888 10.1126/sciadv.aay9842 PMC7527225

[135] M. Bariya , H. Y. Y. Nyein , A. Javey , Nat. Electron. 2018, 1, 160.

[136] A. K. Oktavius , Q. Gu , N. Wihardjo , O. Winata , S. W. Sunanto , J. Li , P. Gao , IEEE Sens. J. 2021, 21, 8861.

[137] A. R. Sharifi , S. Ardalan , R. S. Tabatabaee , S. Soleimani Gorgani , H. Yousefi , K. Omidfar , M. A. Kiani , C. Dincer , T. Naghdi , H. Golmohammadi , Anal. Chem. 2023, 95, 16098. 37882624 10.1021/acs.analchem.3c02044

[138] H. Y. Y. Nyein , M. Bariya , B. Tran , C. H. Ahn , B. J. Brown , W. Ji , N. Davis , A. Javey , Nat. Commun. 2021, 12, 1823. 33758197 10.1038/s41467-021-22109-z PMC7987967

[139] L. Yin , M. Cao , K. N. Kim , M. Lin , J.‐M. Moon , J. R. Sempionatto , J. Yu , R. Liu , C. Wicker , A. Trifonov , F. Zhang , H. Hu , J. R. Moreto , J. Go , S. Xu , J. Wang , Nat. Electron. 2022, 5, 694.

[140] B. Zhang , J. Li , J. Zhou , L. Chow , G. Zhao , Y. Huang , Z. Ma , Q. Zhang , Y. Yang , C. K. Yiu , J. Li , F. Chun , X. Huang , Y. Gao , P. Wu , S. Jia , H. Li , D. Li , Y. Liu , K. Yao , R. Shi , Z. Chen , B. L. Khoo , W. Yang , F. Wang , Z. Zheng , Z. Wang , X. Yu , Nature 2024, 628, 84. 38538792 10.1038/s41586-024-07161-1

[141] J. Uchitel , S. Vanhatalo , T. Austin , Pediatr. Res. 2022, 91, 771. 33859364 10.1038/s41390-021-01497-4

[142] E. K. O'Neill , R. Smith , Eye 2021, 35, 2344. 34290445 10.1038/s41433-021-01663-2 PMC8377028

[143] S. Gao , J. Gong , B. Chen , B. Zhang , F. Luo , M. O. Yerabakan , Y. Pan , B. Hu , Adv. Intell. Syst. 2022, 4, 2200063.

[144] G. Sendić , J. Vasković , The Facial Muscles, Kenhub, https://www.kenhub.com/en/library/anatomy/the‐facial‐muscles (accessed: February 2025).

[145] C. Li , Z. Tan , X. Shi , D. Song , Y. Zhao , Y. Zhang , Z. Zhao , W. Zhang , J. Qi , Y. Wang , X. Wang , Z. Tan , N. Liu , Adv. Sci. 2024, 11, 2406706. 10.1002/advs.202406706 PMC11515898 39206685

[146] Z. Bai , X. Wang , M. Zheng , O. Yue , M. Huang , X. Zou , B. Cui , L. Xie , S. Dong , J. Shang , G. Gong , A. M. Blocki , J. Guo , X. Liu , Adv. Funct. Mater. 2023, 33, 2212856.

[147] W. Wang , Y. Jiang , D. Zhong , Z. Zhang , S. Choudhury , J.‐C. Lai , H. Gong , S. Niu , X. Yan , Y. Zheng , C.‐C. Shih , R. Ning , Q. Lin , D. Li , Y.‐H. Kim , J. Kim , Y.‐X. Wang , C. Zhao , C. Xu , X. Ji , Y. Nishio , H. Lyu , J. B.‐H. Tok , Z. Bao , Science 2023, 380, 735. 37200416 10.1126/science.ade0086

[148] M. Sang , K. Kang , Y. Zhang , H. Zhang , K. Kim , M. Cho , J. Shin , J.‐H. Hong , T. Kim , S. K. Lee , W.‐H. Yeo , J. W. Lee , T. Lee , B. Xu , K. J. Yu , Adv. Mater. 2022, 34, 2105865. 10.1002/adma.202105865 34750868

[149] H. Wang , Y. Mao , D. Ji , L. Wang , L. Wang , J. Chen , X. Chang , Y. Zhu , Chem. Eng. J. 2023, 471, 144674.

[150] S. Yu , S. Wang , L. Zhao , S. Shi , L. Wang , H. Zheng , Inorg. Chem. Commun. 2024, 170, 113219.

[151] S. Lee , D. H. Ho , J. Jekal , S. Y. Cho , Y. J. Choi , S. Oh , Y. Y. Choi , T. Lee , K.‐I. Jang , J. H. Cho , Nat. Commun. 2024, 15, 5974. 39358330 10.1038/s41467-024-49939-x PMC11446925

[152] S. Zhou , G. Park , K. Longardner , M. Lin , B. Qi , X. Yang , X. Gao , H. Huang , X. Chen , Y. Bian , H. Hu , R. S. Wu , W. Yue , M. Li , C. Lu , R. Wang , S. Qin , E. Tasali , T. Karrison , I. Thomas , B. Smarr , E. B. Kistler , B. A. Khiami , I. Litvan , S. Xu , Nat. Biomed. Eng. 2024, 8, 1. 39567702 10.1038/s41551-024-01279-3

[153] J. Li , H. Jia , J. Zhou , X. Huang , L. Xu , S. Jia , Z. Gao , K. Yao , D. Li , B. Zhang , Y. Liu , Y. Huang , Y. Hu , G. Zhao , Z. Xu , J. Li , C. K. Yiu , Y. Gao , M. Wu , Y. Jiao , Q. Zhang , X. Tai , R. H. Chan , Y. Zhang , X. Ma , X. Yu , Nat. Commun. 2023, 14, 5009. 37591881 10.1038/s41467-023-40763-3 PMC10435523

[154] M. Ha , G. S. Cañón Bermúdez , T. Kosub , I. Mönch , Y. Zabila , E. S. Oliveros Mata , R. Illing , Y. Wang , J. Fassbender , D. Makarov , Adv. Mater. 2021, 33, 2005521. 33533129 10.1002/adma.202005521 PMC11469064

[155] M. Matsunaga , J. Hirotani , S. Kishimoto , Y. Ohno , Nano Energy 2020, 67, 104297.

[156] B. Jia , C. Zhang , M. Liu , Z. Li , J. Wang , L. Zhong , C. Han , M. Qin , X. Huang , Nat. Commun. 2023, 14, 5330. 37658051 10.1038/s41467-023-41181-1 PMC10474284

[157] Z. Wang , L. You , V. Pandit , J. Chaudhary , W.‐J. Lee , J. Mei , JACS Au 2024, 4, 2291. 38938807 10.1021/jacsau.4c00254 PMC11200217

[158] C. Xue , Y. Ni , X. Zhao , N. He , J. Li , H. Yu , M. Zhang , X. Liu , B. Wang , J. Sun , X. Han , J. Zhang , J. Sun , Y. Tong , Q. Tang , Y. Liu , Mater. Today Phys. 2023, 36, 101157.

[159] Y. Jin , M. Yu , D. T. Nguyen , X. Yang , Z. Li , Z. Xiong , C. Li , Y. Liu , Y. L. Kong , J. S. Ho , npj Flexible Electron. 2024, 8, 10. 10.1038/s41528-024-00293-4 PMC11619817 39640986

[160] J. Kim , J. Park , Y.‐G. Park , E. Cha , M. Ku , H. S. An , K.‐P. Lee , M.‐I. Huh , J. Kim , T.‐S. Kim , D. W. Kim , H. K. Kim , J.‐U. Park , Nat. Biomed. Eng. 2021, 5, 772. 33941897 10.1038/s41551-021-00719-8

[161] S. Wang , J. Xu , W. Wang , G.‐J. N. Wang , R. Rastak , F. Molina‐Lopez , J. W. Chung , S. Niu , V. R. Feig , J. Lopez , T. Lei , S.‐K. Kwon , Y. Kim , A. M. Foudeh , A. Ehrlich , A. Gasperini , Y. Yun , B. Murmann , J. B.‐H. Tok , Z. Bao , Nature 2018, 555, 83. 29466334 10.1038/nature25494

[162] F. A. Viola , J. Barsotti , F. Melloni , G. Lanzani , Y.‐H. Kim , V. Mattoli , M. Caironi , Nat. Commun. 2021, 12, 5842. 34615870 10.1038/s41467-021-26120-2 PMC8494881

[163] C. Yang , X. Huang , X. Li , C. Yang , T. Zhang , Q. Wu , D. Liu , H. Lin , W. Chen , N. Hu , X. Xie , Adv. Sci. 2021, 8, 2002971. 10.1002/advs.202002971 PMC7967055 33747725

[164] Y. Morimoto , S. Shiu , I. W. Huang , E. Fest , G. Ye , J. Zhu , IEEE Open J. Antennas Propag. 2023, 4, 159.

[165] K. Yamagishi , W. Zhou , T. Ching , S. Y. Huang , M. Hashimoto , Adv. Mater. 2021, 33, 2008062. 10.1002/adma.202008062 34031936

[166] K. N. Paracha , A. D. Butt , A. S. Alghamdi , S. A. Babale , P. J. Soh , Sensors 2019, 20, 177. 31905646 10.3390/s20010177 PMC6983104

[167] P. Qin , Q. Wang , P. Zhang , G. Huang , Q. Li , B. Liu , L. Li , L. Gui , J. Liu , Z. Deng , Int. J. RF Microw. Comput. Aided Eng. 2023, 33, e6369944.

[168] K. Liu , B. Ouyang , X. Guo , Y. Guo , Y. Liu , npj Flexible Electron. 2022, 6, 1.

[169] J. W. Seo , J.‐W. Park , K. S. Lim , J.‐H. Yang , S. J. Kang , Appl. Phys. Lett. 2008, 93, 223505.

[170] H.‐D. Kim , H.‐M. An , Y. Seo , T. G. Kim , IEEE Electron Device Lett. 2011, 32, 1125.

[171] C. H. Ho , J. R. D. Retamal , P. K. Yang , C. P. Lee , M. L. Tsai , C. F. Kang , J.‐H. He , Sci. Rep. 2017, 7, 44429. 28290519 10.1038/srep44429 PMC5349519

[172] J. Sheng , H.‐J. Lee , S. Oh , J.‐S. Park , ACS Appl. Mater. Interfaces 2016, 8, 33821. 27960372 10.1021/acsami.6b11774

[173] K. Song , J. Noh , T. Jun , Y. Jung , H.‐Y. Kang , J. Moon , Adv. Mater. 2010, 22, 4308. 20734383 10.1002/adma.201002163

[174] F. Fleischhaker , V. Wloka , I. Hennig , J. Mater. Chem. 2010, 20, 6622.

[175] L. Jiang , J. Li , K. Huang , S. Li , Q. Wang , Z. Sun , T. Mei , J. Wang , L. Zhang , N. Wang , X. Wang , ACS Omega 2017, 2, 8990. 31457423 10.1021/acsomega.7b01420 PMC6645662

[176] S. Ju , A. Facchetti , Y. Xuan , J. Liu , F. Ishikawa , P. Ye , C. Zhou , T. J. Marks , D. B. Janes , Nat. Nanotechnol. 2007, 2, 378. 18654311 10.1038/nnano.2007.151

[177] E. Song , B. Kang , H. H. Choi , D. H. Sin , H. Lee , W. H. Lee , K. Cho , Adv. Electron. Mater. 2016, 2, 1500250.

[178] J. Xu , S. Wang , G.‐J. N. Wang , C. Zhu , S. Luo , L. Jin , X. Gu , S. Chen , V. R. Feig , J. W. F. To , S. Rondeau‐Gagné , J. Park , B. C. Schroeder , C. Lu , J. Y. Oh , Y. Wang , Y.‐H. Kim , H. Yan , R. Sinclair , D. Zhou , G. Xue , B. Murmann , C. Linder , W. Cai , J. B.‐H. Tok , J. W. Chung , Z. Bao , Science 2017, 355, 59. 28059762 10.1126/science.aah4496

[179] Y.‐Q. Zheng , Z. Bao , ACS Cent. Sci. 2024, 10, 2188. 39735315 10.1021/acscentsci.4c01541 PMC11672543

[180] S.‐H. Kang , J.‐Y. Lee , J.‐H. Park , S.‐G. Choi , S.‐H. Oh , Y.‐C. Joo , S.‐K. Kang , npj Flexible Electron. 2024, 8, 72.

[181] J. Zhang , S. R. Jena , M. Higuchi , ACS Appl. Polym. Mater. 2023, 5, 6950.

[182] Y. Lu , Y. Chen , H. Sun , F. Deng , C. Mei , X. Xu , Q. Wu , H. Xiao , Y. Yue , J. Han , npj Flexible Electron. 2024, 8, 37.

[183] B. Lee , J.‐Y. Oh , H. Cho , C. W. Joo , H. Yoon , S. Jeong , E. Oh , J. Byun , H. Kim , S. Lee , J. Seo , C. W. Park , S. Choi , N.‐M. Park , S.‐Y. Kang , C.‐S. Hwang , S.‐D. Ahn , J.‐I. Lee , Y. Hong , Nat. Commun. 2020, 11, 663. 32005935 10.1038/s41467-020-14485-9 PMC6994701

[184] C. Zhang , A. Khan , J. Cai , C. Liang , Y. Liu , J. Deng , S. Huang , G. Li , W.‐D. Li , ACS Appl. Mater. Interfaces 2018, 10, 21009. 29799181 10.1021/acsami.8b06691

[185] Z. Zhuo , M. Ni , N. Yu , Y. Zheng , Y. Lin , J. Yang , L. Sun , L. Wang , L. Bai , W. Chen , M. Xu , F. Huo , J. Lin , Q. Feng , W. Huang , Nat. Commun. 2024, 15, 7990. 39266527 10.1038/s41467-024-50358-1 PMC11393078

[186] Z. Zhang , W. Wang , Y. Jiang , Y.‐X. Wang , Y. Wu , J.‐C. Lai , S. Niu , C. Xu , C.‐C. Shih , C. Wang , H. Yan , L. Galuska , N. Prine , H.‐C. Wu , D. Zhong , G. Chen , N. Matsuhisa , Y. Zheng , Z. Yu , Y. Wang , R. Dauskardt , X. Gu , J. B.‐H. Tok , Z. Bao , Nature 2022, 603, 624. 35322250 10.1038/s41586-022-04400-1

[187] Y. J. Tan , H. Godaba , G. Chen , S. T. M. Tan , G. Wan , G. Li , P. M. Lee , Y. Cai , S. Li , R. F. Shepherd , J. S. Ho , B. C. K. Tee , Nat. Mater. 2020, 19, 182. 31844282 10.1038/s41563-019-0548-4

[188] C. Xie , X. Zhao , E. W. Y. Ong , Z.‐K. Tan , Nat. Commun. 2020, 11, 4213. 32839475 10.1038/s41467-020-18110-7 PMC7445238

[189] N. Sun , H. Sun , D. Tan , Q. Guo , Z. Zhang , Z. Tao , C. Fang , J. Bu , J. Huang , C. Jiang , Chem. Eng. J. 2023, 469, 143997.

[190] Y. Zhao , H. Liu , Y. Yan , T. Chen , H. Yu , L. O. Ejeta , G. Zhang , H. Duan , Energy Environ. Mater. 2023, 6, 12303.

[191] L. A. Wehner , N. Mittal , T. Liu , M. Niederberger , ACS Cent. Sci. 2021, 7, 231. 33655063 10.1021/acscentsci.0c01318 PMC7908028

[192] T. Liu , X. Chen , E. Tervoort , T. Kraus , M. Niederberger , ACS Appl. Energy Mater. 2021, 4, 6166.

[193] Y. Yang , S. Jeong , L. Hu , H. Wu , S. W. Lee , Y. Cui , Proc. Natl. Acad. Sci. U. S. A. 2011, 108, 13013. 21788483 10.1073/pnas.1102873108 PMC3156205

[194] W. Zhao , M. Jiang , W. Wang , S. Liu , W. Huang , Q. Zhao , Adv. Funct. Mater. 2021, 31, 2009136.

[195] Z. Bai , Y. Xu , C. Lee , J. Guo , Adv. Funct. Mater. 2021, 31, 2104365.

[196] J. Shen , Y. Yang , J. Zhang , W. Lin , H. Gu , ACS Appl. Mater. Interfaces 2024, 16, 46771. 39166375 10.1021/acsami.4c09618

[197] Menge, H. G. , Huynh, N. D. , Choi, K. , Cho, C. , Choi, D. , Park, Y. T. , Adv. Funct. Mater. 2023, 33, 2210571.

[198] R. Meng , Q. Jiang , D. Liu , npj Flexible Electron. 2022, 6, 39.

[199] R. C. Tenent , T. M. Barnes , J. D. Bergeson , A. J. Ferguson , B. To , L. M. Gedvilas , M. J. Heben , J. L. Blackburn , Adv. Mater. 2009, 21, 3210.

[200] N. Cui , Y. Song , C.‐H. Tan , K. Zhang , X. Yang , S. Dong , B. Xie , F. Huang , npj Flexible Electron. 2021, 5, 31.

[201] W. Liu , Z. Du , Z. Duan , L. Li , G. Shen , Nat. Commun. 2024, 15, 5635. 38965218 10.1038/s41467-024-49907-5 PMC11224243

[202] P. Escobedo , M. D. Fernández‐Ramos , N. López‐Ruiz , O. Moyano‐Rodríguez , A. Martínez‐Olmos , I. M. Pérez de Vargas‐Sansalvador , M. A. Carvajal , L. F. Capitán‐Vallvey , A. J. Palma , Nat. Commun. 2022, 13, 72. 35013232 10.1038/s41467-021-27733-3 PMC8748626

[203] B. Hou , D. Yang , X. Ren , L. Yi , X. Liu , Nat. Electron. 2024, 7, 777.

[204] J. H. Lee , H. Kim , J.‐Y. Hwang , J. Chung , T.‐M. Jang , D. G. Seo , Y. Gao , J. Lee , H. Park , S. Lee , H. C. Moon , H. Cheng , S.‐H. Lee , S.‐W. Hwang , ACS Appl. Mater. Interfaces 2020, 12, 21424. 32319751 10.1021/acsami.0c03110

[205] Y. H. Jung , J.‐Y. Yoo , A. Vázquez‐Guardado , J.‐H. Kim , J.‐T. Kim , H. Luan , M. Park , J. Lim , H.‐S. Shin , C.‐J. Su , R. Schloen , J. Trueb , R. Avila , J.‐K. Chang , D. S. Yang , Y. Park , H. Ryu , H.‐J. Yoon , G. Lee , H. Jeong , J. U. Kim , A. Akhtar , J. Cornman , T.‐I. Kim , Y. Huang , J. A. Rogers , Nat. Electron. 2022, 5, 374.

[206] K. K. Kim , M. Kim , K. Pyun , J. Kim , J. Min , S. Koh , S. E. Root , J. Kim , B.‐N. T. Nguyen , Y. Nishio , S. Han , J. Choi , C.‐Y. Kim , J. B.‐H. Tok , S. Jo , S. H. Ko , Z. Bao , Nat. Electron. 2023, 6, 64.

[207] Y. Xu , E. De la Paz , A. Paul , K. Mahato , J. R. Sempionatto , N. Tostado , M. Lee , G. Hota , M. Lin , A. Uppal , W. Chen , S. Dua , L. Yin , B. L. Wuerstle , S. Deiss , P. Mercier , S. Xu , J. Wang , G. Cauwenberghs , Nat. Biomed. Eng. 2023, 7, 1307. 37770754 10.1038/s41551-023-01095-1 PMC10589098

[208] S. Choi , W. Jo , Y. Jeon , S. Kwon , J. H. Kwon , Y. H. Son , J. Kim , J. H. Park , H. Kim , H. S. Lee , M. Nam , E. G. Jeong , J. Bin Shin , T.‐S. Kim , K. C. Choi , npj Flexible Electron. 2020, 4, 33.

[209] Y. Wu , S. S. Mechael , C. Lerma , R. S. Carmichael , T. B. Carmichael , Matter 2020, 2, 882.

[210] S. Hwang , M. Kang , A. Lee , S. Bae , S.‐K. Lee , S. H. Lee , T. Lee , G. Wang , T.‐W. Kim , Nat. Commun. 2022, 13, 3173. 35676280 10.1038/s41467-022-30894-4 PMC9178034

[211] S. Y. Jeong , H. R. Shim , Y. Na , K. S. Kang , Y. Jeon , S. Choi , E. G. Jeong , Y. C. Park , H.‐E. Cho , J. Lee , J. H. Kwon , S. G. Im , K. C. Choi , npj Flexible Electron. 2021, 5, 15.

[212] K. Zhang , X. Shi , H. Jiang , K. Zeng , Z. Zhou , P. Zhai , L. Zhang , H. Peng , Nat. Protoc. 2024, 19, 1557. 38429518 10.1038/s41596-024-00956-6

[213] T. Ding , K. H. Chan , Y. Zhou , X.‐Q. Wang , Y. Cheng , T. Li , G. W. Ho , Nat. Commun. 2020, 11, 6006. 33243999 10.1038/s41467-020-19867-7 PMC7693281

[214] T. Ding , K. H. Chan , Y. Zhou , X. Wang , Y. Cheng , T. Li , G. W. Ho , Nat. Commun. 2020, 11, 6006. 33243999 10.1038/s41467-020-19867-7 PMC7693281

[215] Y. Shi , P. Yang , R. Lei , Z. Liu , X. Dong , X. Tao , X. Chu , Z. L. Wang , X. Chen , Nat. Commun. 2023, 14, 3315. 37286541 10.1038/s41467-023-39068-2 PMC10247702

[216] R. Kaveh , C. Schwendeman , L. Pu , A. C. Arias , R. Muller , Nat. Commun. 2024, 15, 6520. 39095399 10.1038/s41467-024-48682-7 PMC11297174

[217] W. Heng , S. Yin , J. Min , C. Wang , H. Han , E. Shirzaei Sani , J. Li , Y. Song , H. B. Rossiter , W. Gao , Science 2024, 385, 954. 39208112 10.1126/science.adn6471 PMC12168143

[218] J.‐H. Kim , C. Marcus , R. Ono , D. Sadat , A. Mirzazadeh , M. Jens , S. Fernandez , S. Zheng , T. Durak , C. Dagdeviren , Nat. Electron. 2022, 5, 794.

[219] H. Li , H. Gong , T. H. Wong , J. Zhou , Y. Wang , L. Lin , Y. Dou , H. Jia , X. Huang , Z. Gao , R. Shi , Y. Huang , Z. Chen , W. Park , J. Y. Li , H. Chu , S. Jia , H. Wu , M. Wu , Y. Liu , D. Li , J. Li , G. Xu , T. Chang , B. Zhang , Y. Gao , J. Su , H. Bai , J. Hu , C. K. Yiu , et al., Nat. Commun. 2023, 14, 7539. 37985765 10.1038/s41467-023-43189-z PMC10661182

[220] X. Li , C. Luo , Q. Fu , C. Zhou , M. Ruelas , Y. Wang , J. He , Y. Wang , Y. S. Zhang , J. Zhou , Adv. Mater. 2020, 32, 2070162. 10.1002/adma.202000060 32240566

[221] S. Wang , C. M. Fang , Y. Yang , K. Lu , M. Vlachostergiou , L. Yao , in Proc. Augmented Humans International Conf. 2022 , Association For Computing Machinery, New York, NY 2022, pp. 58–67.

[222] R. Kaveti , M. A. Jakus , H. Chen , B. Jain , D. G. Kennedy , E. A. Caso , N. Mishra , N. Sharma , B. E. Uzunoğlu , W. B. Han , T.‐M. Jang , S.‐W. Hwang , G. Theocharidis , B. J. Sumpio , A. Veves , S. K. Sia , A. J. Bandodkar , Sci. Adv. 2024, 10, ado7538. 10.1126/sciadv.ado7538 PMC11305378 39110791

[223] S.‐K. Kim , G.‐H. Lee , C. Jeon , H. H. Han , S.‐J. Kim , J. W. Mok , C.‐K. Joo , S. Shin , J.‐Y. Sim , D. Myung , Z. Bao , S. K. Hahn , Adv. Mater. 2022, 34, 2110536. 10.1002/adma.202110536 PMC10782562 35194844

[224] H. An , X. Wang , Z. Liao , L. Zhang , H. Zhao , Y. Yang , J. Song , Y. Ma , npj Flexible Electron. 2024, 8, 53.

[225] C. Yang , Q. Wu , J. Liu , J. Mo , X. Li , C. Yang , Z. Liu , J. Yang , L. Jiang , W. Chen , H.‐J. Chen , J. Wang , X. Xie , Nat. Commun. 2022, 13, 2556. 35581184 10.1038/s41467-022-29860-x PMC9114010

[226] T. Y. Kim , J. W. Mok , S. H. Hong , S. H. Jeong , H. Choi , S. Shin , C.‐K. Joo , S. K. Hahn , Nat. Commun. 2022, 13, 6801. 36357417 10.1038/s41467-022-34597-8 PMC9649789

[227] J. Zhang , K. Kim , H. J. Kim , D. Meyer , W. Park , S. A. Lee , Y. Dai , B. Kim , H. Moon , J. V. Shah , K. E. Harris , B. Collar , K. Liu , P. Irazoqui , H. Lee , S. A. Park , P. S. Kollbaum , B. W. Boudouris , C. H. Lee , Nat. Commun. 2022, 13, 5518. 36127347 10.1038/s41467-022-33254-4 PMC9489713

[228] L. H. Segura Anaya , A. Alsadoon , N. Costadopoulos , P. W. C. Prasad , Sci. Eng. Ethics 2018, 24, 1. 28155094 10.1007/s11948-017-9872-8

